# A stable subgenomic reporter coronavirus enables transcriptional profiling of bystander cells

**DOI:** 10.1099/jgv.0.002282

**Published:** 2026-06-16

**Authors:** Ciaran Gilbride, Joe Hemsley-Taylor, Catarina Nunes, Rebecca Penn, James Boot, Nima Pieris, Rupa Tripathy, Ziyi Yang, Matthew Hutchinson, Olivia K. Platt, Rachel Ulferts, Richard Mitter, Molly Strom, Nuno B. Santos, David L.V. Bauer, Harriet V. Mears

**Affiliations:** 1 RNA Virus Replication Laboratory, The Francis Crick Institute, London, UK; 2 Viral Vector Core, The Francis Crick Institute, London, UK; 3 Bioinformatics and Biostatistics STP, The Francis Crick Institute, London, UK; 4 School of Biochemistry & Biomedical Sciences, University of Bristol, Bristol, UK; 5 Cell Biology of Infection Laboratory, The Francis Crick Institute, London, UK

**Keywords:** coronavirus, human seasonal coronavirus, HCoV-OC43, reverse genetics, transcriptomics, host response

## Abstract

Insertion of fluorescent reporter genes into viral genomes is a powerful tool for monitoring infection. In coronaviruses, this is commonly achieved by replacing accessory ORFs, thereby deleting endogenous gene functions. An alternative strategy is to manipulate viral RNA synthesis by inserting copies of the viral transcription regulatory sequence (TRS), which drives the transcription of viral subgenomic RNAs. However, coronavirus transcription is tightly regulated, and these modifications frequently disrupt native subgenomic RNA synthesis and attenuate viral growth. Here, we describe a reporter coronavirus that overcomes these limitations. Using human coronavirus (HCoV)-OC43 as a model system, we inserted an mNeonGreen reporter between the Spike and ORF5 coding regions, engineering the TRS and surrounding sequence to minimize off-target effects to transcription. This virus is genetically stable, with WT growth kinetics and unaltered subgenomic RNA transcriptional ratios. We developed a flexible reverse genetics system, which allows rapid cloning and virus recovery, supported by optimized HCoV-OC43 culture conditions for high-titre stock generation, and validated analytical reagents. Our reporter virus enabled sensitive detection and isolation of infected cells, facilitating transcriptomic analyses that distinguish host responses in infected and bystander populations based on active viral translation. We found that transcriptional responses to infection of A549 human lung epithelial cells were predominantly inflammatory, rather than interferon-mediated, and that bystander cells upregulated pathways associated with cytokine response signalling and cell–cell contact sensing. Together, these tools expand the experimental utility of HCoV-OC43, an important seasonal respiratory pathogen and low-containment model for betacoronavirus biology.

## Data availability

All relevant data are within this paper and its supporting information. RNA sequencing data have been deposited in the GEO repository (accession number GSE324533): Mock, bulk (GSM9579192,GSM9579185); WT, bulk (GSM9579191,GSM9579184); dNS2-mNG, bulk (GSM9579182,GSM9579197); ORF4.5-mNG, bulk (GSM9579190,GSM9579183); dNS2-mNG, infected (GSM9579193,GSM9579186); ORF4.5-mNG, infected (GSM9579195,GSM9579188); dNS2-mNG, bystander (GSM9579194,GSM9579187); ORF4.5-mNG, bystander (GSM9579196,GSM9579189).

## Introduction

Coronaviruses are an ongoing threat to global health, with a large animal reservoir and high potential for zoonosis. In the last 25 years, three novel coronaviruses have emerged in the human population, causing epidemics and pandemics of increasing global impact [1,2]. Limited spillover of canine coronaviruses in humans further highlights the persistent zoonotic risk posed by animal coronaviruses [3,4]. In addition to these emergent pathogens, four endemic human coronaviruses (HCoV) circulate worldwide and contribute to the global respiratory disease burden. Seasonal coronaviruses account for 15–30% of common cold cases, of which HCoV-OC43 is the most common [5,9].

**Fig. 1.**
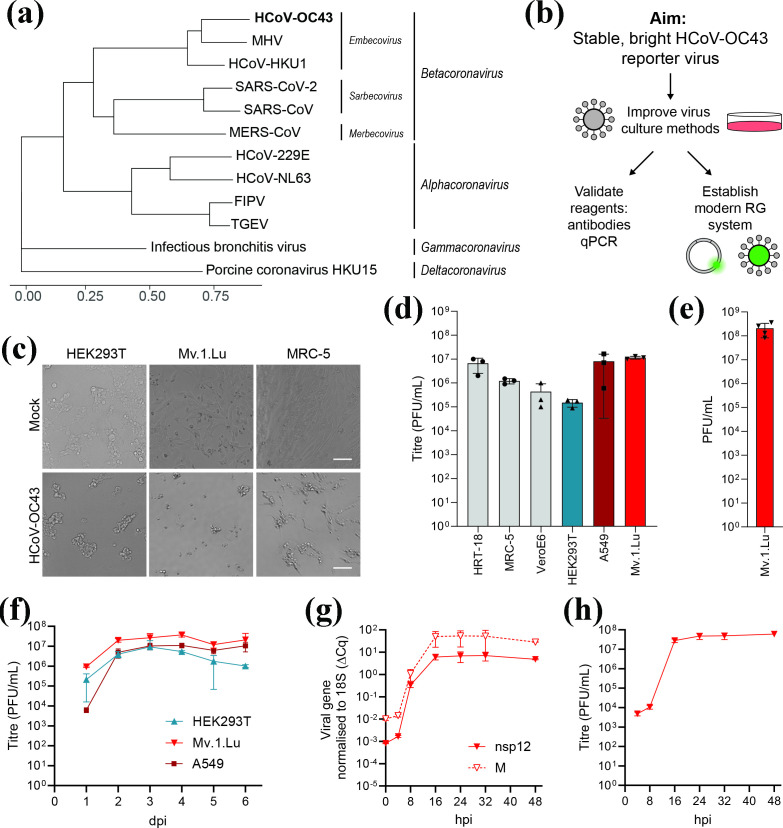
Cell culture conditions for high-titre HCoV-OC43 growth. (**a)** Phylogenetic tree of representative species from the family *Coronaviridae*, based on a codon-level alignment of ORF 1ab. (**b)** Schematic outlining study aims. RG, reverse genetics. (**c)** Light microscopy showing three cell lines (HEK293T, Mv.1.Lu, MRC-5) infected with HCoV-OC43, or mock infected, at an MOI of 0.0001, 5 days post-infection (dpi). Scale bar represents 100 µm. (**d)** Infectious titres of HCoV-OC43 released from the indicated cell lines at 5 dpi in p.f.u. ml^−1^. **e.** Infectious titres of HCoV-OC43 stocks from Mv.1.Lu cells. (**f)** Growth of HCoV-OC43 in Mv.1.Lu, A549 and HEK293T cells infected at an MOI of 0.05. (**g–h)** Growth of HCoV-OC43 in Mv.1.Lu cells infected at an MOI of 3, analysed by RT-qPCR on RNA from cell lysates (**g**) and plaque assay on supernatants (**h**). Viral gene expression (nsp12, solid line, and M, dashed line) was normalized to host 18S RNA (ΔCq). hpi, hours post-infection. Data are means and standard deviations of at least three biological replicates.

While HCoV-OC43 infection typically results in self-limiting upper respiratory tract illness, disease can progress to pneumonia in immunocompromised patients, infants and the elderly [9,12]. Rare cases of central nervous system infiltration have also been reported in mouse models and human patients, leading to viral encephalitis [13,16]. HCoV-OC43 is a *Betacoronavirus*, in the same genus as the highly pathogenic human coronaviruses, SARS-CoV, SARS-CoV-2 and MERS-CoV (Fig. 1a). As such, HCoV-OC43 represents an appealing lower containment model for betacoronavirus biology.

Despite their importance, research into human coronaviruses has historically been constrained by poor growth in cell culture and a lack of effective analytical reagents. While HCoV-HKU1, another seasonal betacoronavirus, is refractory to culture in immortalized cell lines [17,18], HCoV-OC43 can be grown to moderate titres – typically 10^6^ infectious units per millilitre of cell culture supernatant [19,21]. Coronaviruses also possess exceptionally long RNA genomes (~30 kb), often enriched with repetitive AU-rich regions, making them inherently difficult to manipulate using conventional molecular cloning tools [22,28].

Many modern reverse genetics systems rely on transformation-associated recombination (TAR) in *Saccharomyces cerevisiae* to assemble cDNA fragments into stable yeast artificial chromosomes [29,32]. These systems provide superior genetic stability over bacterial artificial chromosomes [25,27,33], while allowing comparatively straightforward genetic manipulation [24,34,36]. Following the emergence of SARS-CoV-2, the development of reverse genetics systems for coronaviruses accelerated dramatically, with a focus on efficient modular cloning strategies [37]. Modern *in vitro* cDNA assembly methods, such as isothermal assembly, Golden Gate cloning and circular polymerase extension reaction (CPER), have now been successfully applied to SARS-CoV-2 [38,39] and other coronaviruses [40,43].

A common goal of viral reverse genetics is to engineer fluorescent or luminescent reporter viruses to monitor and quantify infection. In coronaviruses, tolerance to fluorescent tagging varies across species and genomic loci; for example, while some coronaviruses accommodate reporters at the 5′ end of the genome without substantial fitness costs, others exhibit attenuation [31,44,46]. Reporters are frequently inserted into the structural and accessory gene cassette in the 3′ end of the genome, typically replacing accessory proteins that are dispensable for replication in cell culture [46,50]. However, to avoid deletion of viral genes, it is also possible to engineer dedicated reporter ORFs.

Coronavirus structural and accessory ORFs are expressed from a nested set of subgenomic RNAs (sgRNAs), generated through a process called discontinuous transcription [51]. During negative-strand synthesis, the viral RNA-dependent RNA polymerase complex encounters transcription regulatory sequences upstream of each ORF (TRS-B), which share short, conserved motifs that match the leader transcription regulatory sequence (TRS) (TRS-L) at the 5′ end of the genome. Polymerase template switching from a TRS-B to the TRS-L generates a shorter negative-strand sgRNA, which is subsequently copied into mRNA for the translation of that ORF. Artificial insertion of TRSs in the 3′ end of the genome can therefore drive the transcription of new sgRNAs [52,55] and generate fluorescent reporter viruses [30]. However, the viral transcriptional programme can be disrupted in these viruses, resulting in attenuation and genetic instability [30,54, 55].

To tackle these limitations, we developed a flexible reverse genetics system, using *in vitro* cDNA assembly, allowing reliable virus recovery within 8 days without the need for yeast recombination or *in vitro* transcription steps. We optimized cell culture conditions for HCoV-OC43, finding that mink lung cell lines support high-titre growth, up to 10^8^ p.f.u./ml^−1^. We show that human embryonic kidney (HEK) 293T and lung epithelial (A549) cell lines support productive HCoV-OC43 replication, as well as validating analytical reagents and protocols, to expand the utility of HCoV-OC43 as a research model (Fig. 1b).

Using this system, we generated a novel HCoV-OC43 reporter virus with a dedicated reporter sgRNA, which is genetically stable, with WT growth kinetics and an unaltered sgRNA transcriptional programme. Here, we demonstrate that this virus produces bright fluorescence, compared to insertion of the reporter in place of the NS2 accessory gene, enabling efficient sorting of translationally active infected cells. With this approach, we defined distinct transcriptional programmes in infected and bystander populations by RNA sequencing that reflect coordinated inflammatory cytokine signalling. Together, our work represents a highly tractable toolkit for studying a human coronavirus infection at low containment.

## Methods

### Cell culture and viruses

Cells were maintained in Dulbecco’s Modified Eagle Medium (DMEM), supplemented with 10% FCS and 100 U ml^−1^ penicillin–streptomycin, and routinely tested for mycoplasma by the Francis Crick Institute Cell Sciences facility. Mv.1.Lu cells were a gift from Jonathan Stoye. Vero E6 (Pasteur) cells were a gift from Suzannah Rihn. MRC-5 cells were purchased from the UKHSA culture collection (05072101). Huh-7.5 [56] and Huh-7.5.1 cells [57] were generously provided by Dr Charles M Rice. HEK293T, A549 and HRT-18 cells were provided by the Francis Crick Institute Cell Sciences facility. The HCoV-OC43 isolate was purchased from ATCC (Betacoronavirus 1 VR-1558).

### Infections

Infections were carried out in DMEM supplemented with 2% FCS, and all incubations were carried out at 33 °C. To generate virus stocks, 50–70% confluent Mv.1.Lu cells were inoculated with HCoV-OC43 at a multiplicity of 0.0001 p.f.u. per cell. Cells were incubated for 3–5 days until a cytopathic effect was visible, and cells started to detach from the monolayer. Flasks were frozen at −80 °C, then thawed [58]. Supernatants were clarified by centrifugation at >3,000 ***g*** for 10 min, before aliquoting and storage at −80 °C.

Stocks were titrated by plaque assay on Mv.1.Lu cells. The protocol was adapted from Bracci *et al*. [59], with modifications: briefly, confluent Mv.1.Lu cells in 12-well plates were infected with serial dilutions of HCoV-OC43 in an inoculum volume of 0.25 ml for 60 min. Cells were then overlaid with 1 ml of overlay media: MEM (1 X), FCS (10 %), penicillin–streptomycin (1 %) and Avicel (0.94 %). Dilution of overlay with inoculum results in a final Avicel concentration of 0.75%.

For assessing the stability of fluorescent reporter viruses, Mv.1.Lu cells were infected at a multiplicity of 0.0001 p.f.u. per cell at passage 1. Subsequently, 10 µl of supernatant was passaged blind onto fresh cells every 3–5 days until cytopathic effect was observed, up to passage 5. For all other experiments, multiplicities of infection (MOI) and time points for harvest are indicated in the figures and their respective legends.

Supernatants were harvested and analysed by plaque assay on Mv.1.Lu cells. For RNA extraction, cells were lysed in TRIzol, or supernatants were mixed 1 : 3 with TRIzol LS (Invitrogen). RNA was extracted using the Direct-zol RNA miniprep kit, Direct-zol-96 MagBead RNA kit (Zymo Research), or by phase separation followed by isopropanol precipitation, according to the manufacturer’s protocol. For immunoblotting, cells were harvested in passive lysis buffer (Promega) supplemented with 1% IGEPAL CA-630 (Sigma-Aldrich).

To concentrate viral supernatants, 6×10^7^ Mv.1.Lu cells were infected at an MOI of 0.0001. Supernatants were harvested after 5 days and clarified, overlaid on top of 30% sucrose in 10 mM HEPES pH 7.5, 0.9% NaCl and centrifuged at 100,000 ***g*** for 2 h to pellet virions. Virions were resuspended in HEPES-saline and lysed with passive lysis buffer with 1% IGEPAL CA-630 for immunoblotting.

### Reverse transcription quantitative PCR and reverse transcription PCR

RNA was reverse transcribed using the SuperScript VILO cDNA synthesis kit (Invitrogen) or M-MLV reverse transcriptase (Promega) using random hexamer primers. Primer and probe sequences for HCoV-OC43 M gene have been described [60]. Primers and a probe targeting nsp12 were designed to match the melting conditions and product size of the M primer–probe set to facilitate multiplexing (Table 1). Specificity and linear signal amplification were verified using cDNA standard curves, with PCR amplicons derived from HCoV-OC43 cDNA. For growth curves, data were normalized to cellular 18S rRNA, measured using the Eukaryotic 18S rRNA Endogenous Control (VIC^™^/MGB probe, primer limited) (Applied Biosystems) and expressed as 2^-ΔCq^.

**Table 1.** Primer and probe sequences for reverse transcription qPCR analysis of HCoV-OC43

Target	M	nsp12
Forward	5′-ATGTTAGGCCGATAATTGAGGACTAT-3′	5′-ACGTGGTGTTCCTGTAGTTATAGG-3′
Probe	5′-HEX-CATACTCTGACGGTCACAAT-BHQ-3′	5′-FAM-CAGCCACCATAAAATTTAGTGGT-BHQ-3′
Reverse	5′-AATGTAAAGATGGCCGCGTATT-3′	5′-GGCGGCGTAACATATCATCC-3′
Reference	Vijgen *et al*. [60]	This study

For assessing the genetic stability of fluorescent tags (Fig. 3h, S5D, available with the online Supplementary Material), endpoint PCR was carried out on cDNA from cell supernatants, using Platinum SuperFi PCR Master Mix (Invitrogen) using primers spanning the fluorescent insert (ORF4.5-mNG) or within the mNG coding region (ΔNS2-mNG). PCR cycling conditions were 95 °C for 2 min, followed by 40 cycles of 95 °C for 30 s, 60 °C for 30 s and 72 °C for 60 s, with a final extension of 72 °C for 5 min. PCR products were separated in 1% agarose-TBE gels and visualized using a UVP GelSolo (Analytik Jena).

**Fig. 2.**
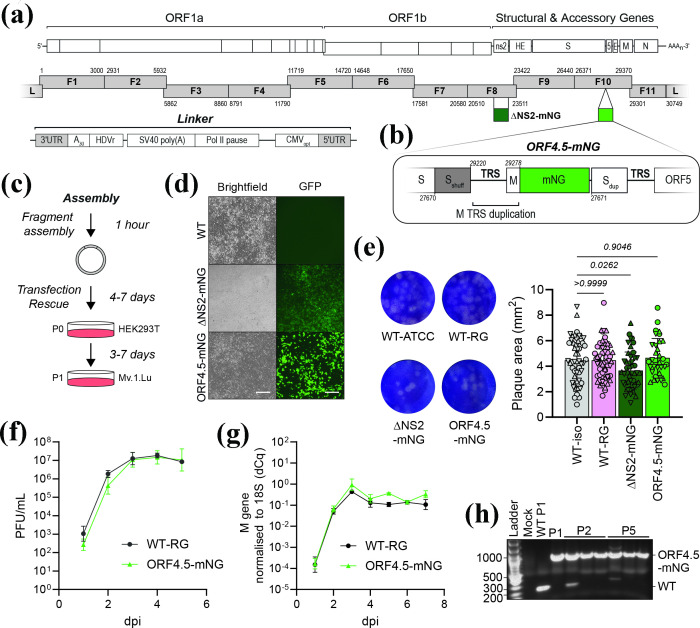
HCoV-OC43 reverse genetics (RG) by *in vitro* assembly from archived fragments. (**a)** Schematic of the HCoV-OC43 genome, fragment and linker design, including a 5′ optimized CMV promoter (CMVopt) and 3′ 30 A tract, Hepatitis D virus ribozyme (HDVr), SV40 poly(A) signal and Pol II pause site. Insertion sites for fluorescent tags (mNeonGreen, mNG) are indicated. (**b)** Details of the ORF4.5 insertion site. The 3′ end of the Spike (S) coding region was codon shuffled to prevent recombination (S_shuff_). The M gene TRS and flanking sequence were inserted upstream of the mNG ORF; the genomic coordinates of the M TRS are indicated above. The WT 3′ end of the S gene was inserted downstream to maintain the sequence context of the ORF5 TRS. (**c)** Scheme for the assembly and rescue of infectious HCoV-OC43. (**d)** Light and fluorescence microscopy showing Mv.1.Lu cells infected with RG-derived HCoV-OC43 viruses (MOI 0.0001, 4 dpi). Scale bars represent 200 µm. (**e)** Plaque morphology of WT (ATCC isolate) and RG-derived HCoV-OC43 viruses (left) and quantification of plaque area (right). Data represent the means and standard deviations of three biological replicates, indicated by different symbols. Data were compared to WT-ATCC by one-way ANOVA, and *P*-values are shown. (**f–g)** Growth of HCoV-OC43 in Mv.1.Lu cells (MOI 0.01), measured by plaque assay (**f**) and reverse transcription qPCR on RNA from cell lysates against the viral M gene, normalized to host 18S rRNA (**g**). Data are means and standard deviations of four (**f**) or three (**g**) biological replicates. (**h)** Agarose gel analysis of PCR amplicons surrounding the ORF4.5-mNG insertion site, amplified from RNA purified from supernatants, from passage 1 (P1), P2 or P5. Three biological replicates of P2 and P5 were analysed.

### Immunoblotting

Cell lysates (5–10 μg total protein) were separated by SDS-PAGE using Any kD precast gels (Bio-Rad) and transferred to a 0.45 μm nitrocellulose membrane by semidry electrotransfer. Membranes were probed with sheep polyclonal anti-Nucleocapsid antiserum (MRC PPU and CVR Coronavirus Toolkit, Sheep No. DA116, 1 : 1,000) [48], rabbit anti-Nucleocapsid (40643-T62, Sino Biological, 1 : 1,000), anti-Spike (PAB21478-100, The Native Antigen Company, 1 : 1,000), anti-SARS-CoV Membrane (ABIN1887462, Antibodies Online, 1 : 200), anti-MHV nucleocapsid J3.3 [61] (1 : 200), anti-GAPDH (AM4300, Invitrogen, 1 : 4,000) and anti-HSP90 (MA5-35624, Invitrogen, 1 : 2,000). IR-dye labelled secondary antibodies (Li-Cor) were visualized using an Odyssey CLx imaging system (Li-Cor).

### Microscopy

**Fig. 3.**
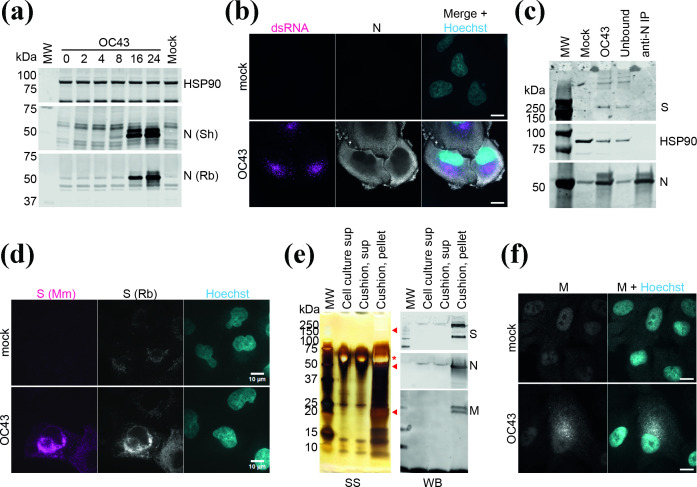
Reagents for analysing HCoV-OC43. (**a)** Western blot of Mv.1.Lu cells infected with HCoV-OC43 at an MOI of 10 p.f.u. per cell and harvested at the indicated time points, probed with two anti-nucleocapsid (N) antibodies, derived from sheep (Sh) polyclonal antiserum from the MRC PPU and CVR coronavirus toolkit and a commercial Rabbit (Rb) polyclonal antiserum. Host HSP90 is included as a loading control. (**b)** Immunofluorescence microscopy of A549 cells infected with HCoV-OC43, stained with Sheep anti-N and anti-dsRNA, a marker of RNA replication. (**c)** Immunoprecipitation (IP) of lysates from HCoV-OC43-infected cells (MOI 3, 24 hpi) using Sheep anti-N and probed with Sheep anti-N and anti-Spike (S). Host HSP90 is included as a loading control. (**d)** Immunofluorescence microscopy of HCoV-OC43-infected A549 cells, stained with mouse monoclonal (Mm) or rabbit polyclonal (Rb) anti-S. (**e)** Silver stain (SS) and Western blotting (WB) of virions purified by ultracentrifugation through a 30% sucrose cushion. The supernatant (sup) before and after the sucrose cushion, and the pellet, containing purified virions, are shown. Viral structural proteins are indicated by red arrowheads and BSA from cell culture medium by a red asterisk. (**f)** Immunofluorescence microscopy of HCoV-OC43-infected A549 cells stained with anti-SARS-CoV M. Scale bars represent 10 µm.

To image cytopathic effect (Figs 1c and 2), cells were imaged using the ZOE Fluorescent Cell Imaging System (Bio-Rad). Fluorescent viruses (Figs 3d and 5) were imaged using an EVOS M5000 Imaging System (Invitrogen).

A549 cells grown on coverslips were infected with HCoV-OC43 or mock-infected and fixed at 20 hpi in 4% formaldehyde (Sigma) for 20 min. Cells were permeabilized with 0.1% Triton X-100 (Sigma) for 3 min and blocked in 3% BSA (Sigma, A9647) for 30 min. Primary antibodies are listed under the ‘Immunoblotting’ section; additionally, mouse anti-dsRNA J2 (absolute antibodies, AB01299-2.0), mouse anti-N clone 542-7D (Merck, MAB9013) and mouse anti-S clone 29 (Sino Biological, 68086-MM29) were used. All primary antibodies were diluted 1 : 1,000 and were applied for 1 h at room temperature, followed by donkey anti-rabbit or anti-mouse Alexa Fluor Plus 555 or 647 secondary antibodies (Thermo Fisher) for 45 min. Nuclei were stained with Hoechst 33342 (Sigma). Coverslips were mounted in ProLong Glass (Thermo Fisher) and imaged on a VTI-SIM microscope (VisiTech). Images were processed in Fiji/ImageJ [62,63].

### Plasmids

An archiving vector, pCLVR, was designed based on a pUC vector background, with the addition of bidirectional *Escherichia coli* transcriptional terminators flanking the cloning site, to minimize read-in or read-out transcription from inserts. The cloning site consists of 5′ and 3′ BsaI and BsmBI sites, which facilitate scarless excision of inserts. The pCLVR vector contains an mRFP1_Magenta chromoprotein [64] between the cloning sites, which is excised upon fragment insertion, enabling colour-based screening of bacterial colonies.

Primers were designed across the HCoV-OC43 genome, using the ATCC reference strain, split into 11 fragments that overlap by 70 nt to allow genome assembly. Sequences were amplified from cDNA derived from HCoV-OC43-infected cells and assembled individually or in pairs into the pCLVR archive plasmid. Finally, a linker plasmid was synthesized (Genscript), containing a 30-nt poly-A sequence, followed by a hepatitis delta virus ribozyme, the SV40 terminator and a PolII pause site; the linker contains a modified CMV promoter sequence, with a focussed initiator consensus sequence [65] to drive specific initiation at the 5′ end of the HCoV-OC43 genome. These sequences are flanked by ~75 nt of the HCoV 3′ and 5′ UTRs, respectively, to facilitate assembly into a single circular plasmid. Finally, fluorescent reporters were codon optimized to match the low GC content of coronavirus genomes and were synthesized and cloned into the pCLVR archive vector (Genscript).

### HCoV-OC43 assembly and rescue

For assembly, fragments were PCR amplified from the pCLVR archive plasmid using Platinum SuperFi PCR Master Mix (Invitrogen). PCR products were purified using the NucleoSpin Gel and PCR Clean-up Kit (Macherey-Nagel), and equimolar amounts (typically 200 ng DNA per 6 kb fragment with 50 ng linker) were assembled using the NEBuilder HiFi DNA Assembly Master Mix (New England Biolabs).

Assembly reactions were stored or transfected directly into HEK293T cells, without further purification, using Lipofectamine 3000 (Invitrogen). After incubation overnight, the media were exchanged for fresh DMEM supplemented with 2% FCS. Cells were then incubated for a further 3 to 7 days at 33 °C. Passage 0 supernatants were amplified on Mv.1.Lu cells to generate P1 viral stocks.

A detailed protocol for HCoV-OC43 reverse genetics, including plasmid and oligonucleotide sequences, cell culture protocols and analytical reagents, is provided as a supplementary protocol.

### Cell sorting

To isolate mNG-positive (infected) and mNG-negative (bystander) populations, 90–100% confluent A549 cells were inoculated with either HCoV-OC43-WT, HCoV-OC43-ΔNS2-mNG or HCoV-ORF4.5-mNG at an MOI of 1 p.f.u. per cell. After incubation for 24 h at 33 °C, cells were trypsinized, centrifuged and incubated in Zombie NIR^™^ viability dye (Sony Biotechnology, 1 : 1,000).

Cells were sorted using a BD FACSAria III cell sorter (BD Biosciences). The thresholds for detection of mNG-positive cells were set using a mock-infected control. Cell populations were sorted into collection tubes containing 2% foetal calf serum and 0.5 µl RNasin (Promega). Flow cytometry data were analysed in FlowJo software (v10.10).

### RNA sequencing

For both bulk RNA-seq and sorted RNA-seq, RNA extractions were performed by lysing cells in TRIzol (Invitrogen). RNA was extracted using the Direct-zol RNA Miniprep Kit or Direct-zol RNA Microprep Kit (Zymo Research). The Genomics Science Technology Platform at the Francis Crick Institute prepared reverse-stranded sequencing libraries from 40 ng of RNA using the Watchmaker mRNA Library Prep Kit (modules K0105 and K0078), following the manufacturer’s protocols. Paired-end sequencing (100 bp) was performed on an Illumina NovaSeq X platform, with an average depth of 25 million reads per sample.

### Data analysis

Paired-end bulk RNA sequencing reads were processed using the nfcore/rnaseq pipeline (v3.10.1). The pipeline utilized STAR [66] for read alignment to the human reference genome (GRCh38.95 from Ensembl release 95, with corresponding GTF annotation v95), and quantification was performed using RSEM [67]. Read count matrices from the nfcore pipeline were imported into R for differential expression analysis using DESeq2 [68]. The DESeq2 workflow included the following: filtering of low-abundance genes to improve statistical power and reduce multiple testing burden, estimation of size factors for normalization across samples, dispersion estimation and shrinkage for improved estimates of fold-changes and multiple hypothesis testing correction using the Benjamini–Hochberg false discovery rate method. The frequency of single-nucleotide polymorphisms compared to the Betacoronavirus 1 VR-1558 reference genome was determined using LoFreq [69].

Gene set enrichment analysis (GSEA) was performed using clusterProfiler (v4.14.6) [70] using Fast GSEA v1.32.4 [71] against curated Biological Pathway and Molecular Function databases. A 60% simplify threshold was applied to collapse redundant terms. Analyses were conducted with organism-specific annotation databases (org.Hs.eg.db for human samples). Heatmaps were visualized using pheatmap (v1.0.13 https://github.com/raivokolde/pheatmap). Gene sets for heatmaps were based on GSEA terms enriched in HCoV-OC43-ORF4.5-infected or bystander populations.

### sgRNA analysis

A subset of viral-mapping reads was further processed through a pipeline to identify discontinuous viral transcripts, including canonical sgRNAs. The analysis was conceptually analogous to detecting spliced eukaryotic transcripts. Raw paired-end sequencing reads were processed using TrimGalore! (v0.6.10) to remove adapters and low-quality sequences, and reads with a minimum length of 30 bp were retained. Overlapping R1 and R2 reads were merged using FLASH (v1.2.11) [72] with a minimum overlap of 18 bp and a maximum overlap difference of 0.25. Merged reads were combined with unmerged R1 reads, which remained on the forward (R1) strand. For unmerged R2 reads, reverse complementation was performed using the FASTX Toolkit (v0.0.14, http://hannonlab.cshl.edu/fastx_toolkit/) before merging. Finally, all merged sequences (which aligned to the original R1 negative strand) were reverse-complemented to the positive strand orientation for viral genome alignment.

For each viral variant, a joint reference genome was constructed by concatenating the human reference genome (GRCh38.95 from Ensembl release 95) with the corresponding viral genome sequence and annotations, and STAR indices (v2.7.11a) were generated [66]. Separate STAR indices were also constructed for each viral genome alone; the --genomeSAindexNbases parameter was set to 6 for viral-only indices due to the smaller genome size.

Preprocessed reads were aligned to each joint host–viral reference genome using STAR, and reads mapping to viral chromosomes were extracted. Reads mapping to viral sequences were re-aligned to viral-only genomes using STAR with stringent parameters optimized for detecting chimeric reads (mapping uniquely to two regions of the genome, with a single breakpoint), corresponding to discontinuous coronavirus transcripts. Key parameters included the following: --outFilterType BySJout, --alignSJoverhangMin 20, --outSJfilterOverhangMin 20 20 20 20, --outSJfilterCountUniqueMin 1 1 1 1, --outSJfilterCountTotalMin 1 1 1 1, --outSJfilterDistToOtherSJmin 0 0 0 0, --scoreGapNoncan 0, --scoreGapGCAG 0, --scoreGapATAC 0, --alignSJstitchMismatchNmax 0 0 0 0, bases --outFilterMatchNmin 40, --outFilterMismatchNoverLmax 0.1, --alignEndsType Local, --outSAMmultNmax 1, --alignSJDBoverhangMin 20.

BAM files from viral-specific alignments were processed using GenomicAlignments in R [73] to extract alignment information. The number of chimeric and nonchimeric alignments was counted for each sample. A background measure of nonchimeric (i.e. genomic) reads was defined for each sample to account for variation in viral RNA abundance across samples. The genomic background region was defined as a 1 kb window centred on the midpoint between the start coordinate of the S sgRNA intron and the start of the ORF1ab gene body. Nonchimeric reads that overlapped this region were counted as ‘genomic’ alignments and used as a normalization factor for subsequent analyses. Transcript junctions were extracted from chimeric alignments using the ‘summarizeJunctions()’ function, which identified all unique junction coordinates and their read depths.

Junctions were classified as either canonical (donor and acceptor sites match coordinates in the annotated sgRNA GTF), alternative (only one donor or acceptor site match) or defective (neither donor nor acceptor site match). Within the canonical class, junctions were further subclassified by their corresponding sgRNA gene identity (e.g. E, M and N). Defective junctions were subclassified based on the size of their genomic span (gap size).

### Phylogenetic tree

The ORF1ab sequence was extracted from representative coronavirus genome sequences (NC_005831.2, NC_039208.1, NC_002645.1, NC_006577.2, NC_004718.3, NC_048217.1, NC_019843.3, NC_006213.1, NC_045512.2, NC_048213.1, NC_038861.1, NC_002306.3). Protein alignments were made using MAFFT [74], and trees were constructed using FastTree [75], visualized using ape (doi:10.1093/bioinformatics/bty633) and ggtree [76].

## Results

### Cell culture models for high-titre HCoV-OC43 growth

We first sought to identify a suitable cell line for high-yield HCoV-OC43 propagation to support downstream reverse genetics development. We infected a panel of cells at a low multiplicity of infection (0.0001 p.f.u. ml^−1^) with HCoV-OC43 (ATCC Betacoronavirus 1, VR-1558). These consisted of MRC-5 human fibroblasts and HRT-18 human rectal tumour cells, which are frequently used for seasonal coronavirus culture, as well as VeroE6, which were recently reported as an optimal cell line for HCoV-OC43 growth [20]. We included two further commonly used human cell lines, HEK293T and A549, as well as a mink lung cell line (Mv.1.Lu), which has previously been used to assess HCoV-OC43 growth by plaque assay [59].

After 4 days, we observed substantial cytopathic effect (CPE) in infected MRC-5, as expected, as well as CPE in both Mv.1.Lu cells and HEK293T cells (Fig. 1c). When supernatants were analysed by plaque assay, we found high infectious titres from both Mv.1.Lu and A549 cells at 5 days post-infection (dpi) (Fig. 1d). Stocks from Mv.1.Lu cells exceeded 10^8^ p.f.u. ml^−1^ (Fig. 1e), and amplicon sequencing showed no consensus-level deviations from the reference sequence. As such, Mv.1.Lu cells represent a new cell model for high-titre HCoV-OC43 growth.

We then examined the kinetics of HCoV-OC43 growth in Mv.1.Lu cells, HEK293T and A549. Cells were infected at a low (0.05 p.f.u. per cell) multiplicity and incubated for up to 6 days before supernatants were analysed by plaque assay and reverse transcription quantitative PCR (qPCR). A primer-probe assay has been described previously [60], which binds within the M gene coding region. We used this as a basis to design a second primer-probe set that binds within nsp12 to measure genomic RNA replication. Melting temperatures were matched to the M primer–probes to facilitate multiplexing, and primer efficiency and specificity were verified using cDNA standard curves (Fig. S1B–C).

We observed rapid replication in Mv.1.Lu cells, with high titres (>10^6^ p.f.u. ml^−1^) even at 1 day post-infection and peak titres at 2–4 days (Fig. 1f). Titres from Mv.1.Lu cells were consistently higher than either A549 or HEK293T cells. Infectious titres correlated with genome replication, which increased rapidly between 1 and 2 dpi, and then waned (Fig. S1D). We then infected Mv.1.Lu cells at a high (3 p.f.u. per cell) multiplicity and monitored replication over 48 h. Genome replication occurred between 4 and 16 h post-infection (Fig. 1g), while infectious titres increased from 8 to 16 h post-infection, lagging behind genome replication by ~4 h (Fig. 1h). Titres plateaued by 24 h post-infection, when substantial CPE was observed.

In parallel, we determined the growth of two seasonal alphacoronaviruses, HCoV-229E and HCoV-NL63, in a wide panel of cell lines (Fig. S2). We found that Mv.1.Lu cells did not support high-titre growth of these viruses; rather, Huh7.5.1 cells supported HCoV-229E growth (up to 10^7^ p.f.u. ml^−1^, Fig. S2D), while LLC-MK2 cells supported low-titre HCoV-NL63 growth (10^4^ p.f.u. ml^−1^, Fig. S2E), consistent with standard protocols.

### Reagents for HCoV-OC43 analysis

In addition to our multiplexed reverse transcription qPCR assay, we evaluated reagents for immunodetection of HCoV-OC43 proteins. We tested two anti-N antibodies: a rabbit polyclonal antibody and a sheep-derived polyclonal antibody [48]. Both antibodies detected N expression in Mv.1.Lu cells, following high multiplicity infection (3 p.f.u. per cell), with robust detection at 16 and 24 h, and as early as 8 h post-infection for the sheep polyclonal antibody (Fig. 2a). The sheep polyclonal also detected a second band with slower migration, consistent with phosphorylated N.

Immunofluorescence microscopy confirmed specific N staining with both antibodies, with minimal background in mock-infected controls (Fig. 2b, Figure S3A, B). Consistent with other coronavirus N proteins [48,77], HCoV-OC43 N localized predominantly to the cytoplasm and cell periphery. Finally, immunoprecipitation using the sheep anti-N antibody successfully enriched N from infected cell lysates (Fig. 2c). Spike protein was also detectable in infected cell lysates by immunoblotting (Fig. 2c) and in virions purified by sucrose cushion ultracentrifugation (see Fig. 2e) using a rabbit polyclonal antibody. By immunofluorescence microscopy, the rabbit polyclonal showed background punctate staining in mock-infected cells, consistent with mitochondria, while a mouse monoclonal antibody had low background in mock infection; both showed perinuclear staining in infected cells (Fig. 2d), consistent with S accumulation in the endoplasmic reticulum (ER).

No commercial antibodies specific to HCoV-OC43 membrane (M) protein were available. Given the moderate sequence conservation across betacoronavirus M proteins, we hypothesized that some antibodies raised against heterologous M proteins might recognize HCoV-OC43 M. We, therefore, screened a panel of antibodies raised against M proteins from related betacoronaviruses for potential cross-reactivity. A monoclonal antibody against mouse hepatitis virus (MHV) M [61] failed to detect HCoV-OC43 M by immunoblot (Fig. S3C). In contrast, a polyclonal antibody against SARS-CoV M weakly detected a band consistent with M in infected cell lysates at 24 h post-infection (Fig. S3C). However, we noticed considerable background signal in mock-infected cells, precluding reliable detection of M in whole cell lysates (Figure S3D, lane 1). To reduce this background and enrich for viral structural proteins, virions were purified by sucrose cushion ultracentrifugation, which enabled definitive detection of M by western blot (Figs 2e and S3D, lane 4).

Finally, by immunofluorescence microscopy, the anti-SARS-CoV-M antibody displayed nonspecific nuclear staining in mock-infected controls, but additional punctate perinuclear staining specific to infected cells (Fig. 2f), consistent with the localization of other coronavirus M proteins [78,80]. Together, these data indicate that, although background signal limits sensitivity, cross-reactive antibodies can be used to detect HCoV-OC43 M protein in purified virions and for imaging experiments.

### Generation of fluorescent reporter HCoV-OC43 viruses

We next developed a reverse genetics system for HCoV-OC43, leveraging modern *in vitro* assembly methods for rapid, high-fidelity viral assembly. The viral genome was divided into ten overlapping ≈3 kb fragments (F1-10), along with a terminal 1.5 kb fragment (F11) encompassing the genomic 3′ end (Fig. 3a and b). A linker fragment (L) was designed to include a CMV promoter, to drive transcription of the viral genome in mammalian cells; for 3′ end processing, L encodes a poly-A tract and hepatitis delta virus ribozyme, followed by an SV40 polyadenylation signal and a Pol II transcriptional terminator (Fig. 3a). Genome fragments were amplified from cDNA derived from HCoV-OC43-infected cells and cloned in pairs into a modified pUC vector, termed pCLVR. To minimize bacterial toxicity associated with leaky expression of viral sequences, the cloning site in pCLVR is flanked by bi-directional *E. coli* transcriptional terminators (Figure S4A).

This modular design results in a six-plasmid system that supports full-length genome assembly, to generate a single circular DNA containing the full-length HCoV-OC43 genome, with a 5′ promoter and 3′ polyadenylation and terminator elements. We found that CPER assemblies were prone to precipitation and could not be stored prior to transfection, while isothermal assembly methods (e.g. Gibson or HiFi) were more stable and amenable to storage. Assembled genomes were transfected into HEK293T cells using liposomes and incubated for 4–7 days, until CPE was observed. Passage 0 supernatants were amplified on Mv.1.Lu cells for a further 3–7 days to generate a P1 stock (Fig. 3c). For the WT virus, titres of 10^8^ p.f.u. ml^−1^ could be obtained within 10 days of initial assembly.

We designed two fluorescent reporter viruses (Fig. 3a and b). First, we replaced the HCoV-OC43 NS2 ORF, with an mNeonGreen (mNG) reporter to create HCoV-OC43-ΔNS2-mNG. NS2 is a phosphodiesterase, which antagonizes the host antiviral factor RNaseL [81,82] and is dispensable for replication in cell culture [83]. The mNG coding sequence was codon-optimized to match the low GC content of the HCoV-OC43 genome. Fluorescence could be reliably observed at 4 days post-transfection, indicating successful rescue (Figure S4B); likewise, when Mv.1.Lu cells were infected at a low multiplicity of infection, the majority of cells showed green fluorescence at 4 dpi (Fig. 3d). HCoV-OC43-ΔNS2-mNG viruses displayed slightly delayed growth kinetics compared to WT virus (Figure S4C) and produced slightly smaller plaques (20% reduction in area relative to WT, Fig. 3e), indicative of a minor loss of fitness. Nevertheless, after two (P2) and five (P5) passages in Mv.1.Lu cells, the mNG insert was maintained, determined by endpoint RT-PCR on RNA extracted from viral supernatants (Figure S4D).

To generate a reporter virus that preserves native viral protein function, we engineered a novel sgRNA to express mNG without disrupting endogenous ORFs. We modelled our synthetic TRS on the well-expressed M gene TRS, incorporating 28 nt upstream and 24 downstream of the TRS core sequence (UCCAAAC), including predicted RNA structural elements. This region included the first 21 nt of the M ORF, followed by the mNG reporter. To preserve the genomic context required for downstream TRS activity (i.e. ORF5), we appended the native 3′ end of the S gene after the mNG stop codon. To reduce the risk of homologous recombination between the duplicated S sequences, the upstream region was codon-shuffled while preserving the amino acid sequence. The resulting reporter sgRNA was designated ORF4.5 (Fig. 3b).

**Fig. 4.**
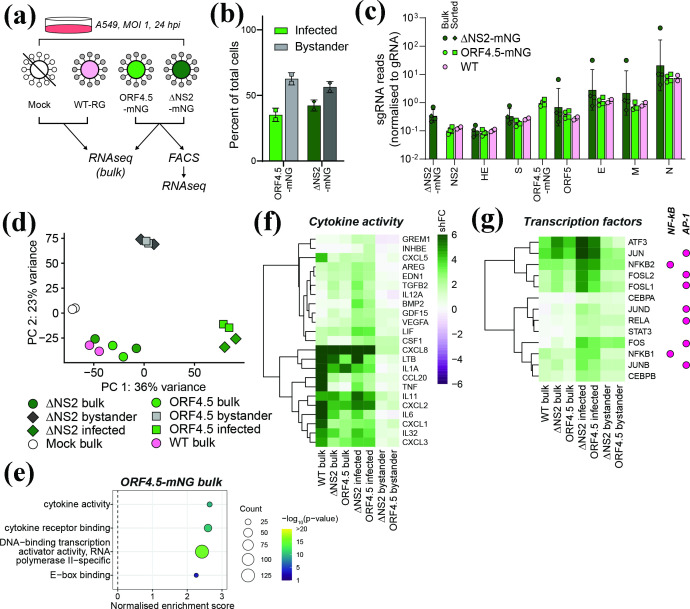
HCoV-OC43-ORF4.5-mNG transcription is comparable to WT and triggers an inflammatory cytokine response in infected A549 cells. (**a)** Schematic showing the experimental plan for transcriptomic analysis. A549 cells were infected at an MOI of 1 and harvested after 24 h. RNA was either extracted from the whole cell population (bulk), or cells were sorted by mNG fluorescence before RNA extraction from infected (mNG positive) or bystander (mNG negative) populations. (**b)** Percentage of infected (green) and bystander (grey) cells after cell sorting. (**c)** Expression of viral sgRNA, determined by mapping chimeric reads over the leader-TRS-body junction, normalized to genomic (gRNA) read counts. N.b. in ΔNS2-mNG, the NS2 TRS drives expression of mNG. (**d)** Principal component (PC) analysis of transcriptomics data. (**e)** Gene set enrichment analysis of differentially expressed genes in bulk ORF4.5-mNG-infected cells, based on molecular function. Functions are ranked by normalized enrichment score and coloured by significance. (**f–g)** Heatmaps showing cytokine activity (**f**) and induction of pro-inflammatory transcription factors (**g**), coloured by shrunk log_2_ fold change (shFC) in expression compared to mock cells.

HCoV-OC43-ORF4.5-mNG was rescued and grew to titres comparable to WT-RG virus, with similar growth kinetics (Fig. 3f and g) and no changes to plaque phenotype (Fig. 3e). We observed brighter fluorescence compared to HCoV-OC43-ΔNS2-mNG (Fig. 3d), indicating the synthetic ORF is well expressed. Upon serial passage, the ORF4.5 insert was maintained up to P5 (Fig. 4h), and the fluorescent signal remained bright (Figure S4E, F). Together, these results show that the HCoV-OC43-ORF4.5-mNG is a stable, bright reporter virus with WT-like growth kinetics.

### Transcriptomic analysis of sorted infected and bystander cells

Finally, we aimed to understand how different populations of cells respond to infection by profiling the host transcriptional response to HCoV-OC43 in both infected and bystander cells. Differentiating between infected and bystander cells usually relies on the detection of cell-surface viral antigens [84], which limits analysis to cells at later stages of infection. By contrast, our fluorescent reporters should facilitate sensitive detection and sorting of infected cells even early in the viral lifecycle, without the need for antibody staining. We infected A549 cells with HCoV-OC43-ΔNS2-mNG, HCoV-OC43-ORF4.5-mNG or WT virus (Fig. 4a). A549 cells are a human lung carcinoma cell line, which can sense and respond to viral infection and support productive HCoV-OC43 replication (Figure S5A). After 24 h, RNA was extracted from the whole cell population (bulk) or following live-cell fluorescence-activated cell sorting.

For both reporter viruses, we saw effective separation of productively infected, mNG-positive cells from the rest of the population (Figs 4b and 6). These mNG-negative cells were, therefore, designated as the bystander cell population. Consistently, we saw an enrichment of viral reads in infected cells following sorting, increasing from ~50% of total RNA in bulk cell populations to ~70% (Figure S5B). When we analysed viral transcription, we found that viral subgenomic RNA expression was comparable between our WT and reporter viruses (Fig. 4c). Importantly, the insertion of ORF4.5 did not affect the transcription of up- or downstream sgRNAs, while ORF4.5 itself was expressed to levels comparable to the M sgRNA. Likewise, we observed no consensus-level mutations in HCoV-OC43-ORF4.5-mNG, and a low frequency of single-nucleotide polymorphisms, relative to the reference genome, supporting the genetic stability of this virus and the fidelity of our reverse genetics system (Figure S5C). Bystander cells showed 5–10% of reads mapping to the viral genome (Figure S5B). These cells may, therefore, represent a heterogeneous population of truly uninfected cells, with abortively infected cells and/or cells at an early stage of infection, before substantial viral protein production.

We then assessed host responses to infection. RNA-seq from bulk cell populations, infected with WT or fluorescent reporter viruses, showed highly similar transcriptional signatures, which were distinct from mock-infected controls, as shown by principal component analysis (Fig. 4d). In contrast, RNA-seq of FACS-sorted populations revealed that infected and bystander cells formed separate clusters, distinct from both mock and bulk samples, indicating that sorting effectively isolated transcriptionally unique cell populations (Fig. 4d).

Analysis of bulk populations showed robust upregulation of inflammatory response genes following HCoV-OC43 infection (Fig. 4e and f, Figure S7A–E). Infected cells displayed increased expression of multiple cytokines, including proinflammatory interleukins, and chemokines associated with neutrophil recruitment (Fig. 4f). Indeed, among the most strongly upregulated transcripts in infected cells were those encoding the neutrophil chemoattractant CXCL8 and the proinflammatory cytokines IL-1A and IL-6. We also observed induction of transcription factors, consistent with an early transcriptional response to cytokine signalling (Fig. 4g). For example, we observed induction of NF-KB and AP-1 genes, which are typically induced in response to TNF and IL-1A. This may be counterbalanced by concomitant upregulation of anti-inflammatory factors such as TNFAIP3, which regulates NF-KB signalling to prevent excessive inflammation, and dual-specificity phosphatases, which control MAP-kinase activity (Figure S7G). In contrast, we did not observe a classical IFN response in infected cells (Figure S7H). Instead, we saw upregulation of TGF-β signalling (Figure S8A), which may contribute to suppression of IFN responses [85,86].

**Fig. 5.**
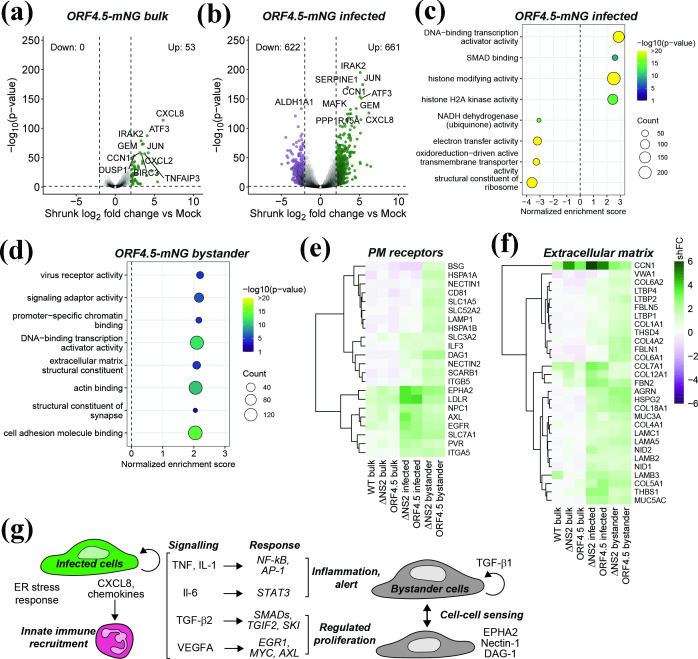
Sorting infected cells enriches for differentially expressed transcripts and reveals a unique transcriptional response in bystander cells. (**a–b)** Volcano plots showing differentially expressed genes in HCoV-ORF4.5-mNG-infected cells, either from the bulk population (**a**) or after sorting (**b**), expressed as shrunk log_2_-fold change over mock-infected cells. Significantly up- or downregulated genes (shrunk log2 fold change>±2, *P*-value <0.05) are coloured, and the top ten most significant genes are labelled. (**c–d)** Gene set enrichment analysis of differentially expressed genes in sorted, ORF4.5-mNG-infected cells (**c**) or bystander cells (**d**), based on molecular function. Functions are ranked by normalized enrichment score (NES) and coloured by significance. (**e–f)** Heatmap showing genes associated with plasma membrane (PM) receptors (**e**) and extracellular matrix structural components (**f**), coloured by shrunk log2 fold change (shFC) in expression compared to mock cells. (**g)** Model for transcriptional responses in infected and bystander cells. Infected cells have a strong transcriptional response to HCoV-OC43, including secretion of pro-inflammatory cytokines and chemokines, as well as an ER stress response and downregulated metabolism. Bystander cells respond by upregulating pro-inflammatory pathways, including NF-κB, counterbalanced by mild anti-inflammatory and pro-proliferative signalling. Bystander cells upregulate factors associated with cell–cell sensing and wound repair.

Sorted, infected cells exhibited a substantially larger number of differentially expressed genes compared to sequencing the bulk cell population (compare Fig. 5a and b, S7B and S7C). In addition to inflammatory signalling, we could also detect a greater degree of transcriptional remodelling (Fig. 5c). In particular, there is upregulation of a subset of histone remodelling factors, transcriptional activators and transcriptional repressors, supporting selective and regulated reprogramming of transcriptional output.

Transcriptional responses in the bystander (mNG-negative) cell population were more muted than in infected cells: although many genes were significantly upregulated (Figure S8B, C), the average fold change relative to mock cells was ≈2-fold, compared to ≈2.5-fold in infected cells (Figure S8D). In addition to genes associated with cytokine-responsive pathways (Figs 4g, 5d and S8C), bystander cells upregulated a subset of TGF-*β* signalling genes, which was distinct from infected cells, including both positive and negative regulators of TGF-*β* signalling (Figure S8A). In particular, infected cells showed higher expression of the *TGFB2* isoform, while bystander cells preferentially expressed the *TGFB1* transcript, as well as transcripts encoding regulatory factors like SKI, which represses TGF-*β* signalling. Bystander cells also displayed transcriptional changes consistent with alterations in plasma membrane composition and lipid-associated processes, including upregulation of genes encoding cell surface receptors and proteins involved in lipid metabolism that were not significantly induced in infected cells (Fig. 5d and e). These included growth factor receptors and sensors of extracellular matrix disruption (Fig. 5f). There was also upregulation of genes encoding actin cytoskeletal components, which was distinct from infected cells (Figure S8E). Together, this indicates sensing of local cell damage and cell migration in response to local damage.

Finally, we detected extensive downregulation of host transcripts in sorted, infected cells (Fig. 5c), a feature largely absent in bulk data. These downregulated genes that were enriched encoded components of aerobic respiration and mitochondrial metabolism, consistent with a shift away from oxidative metabolism during HCoV-OC43 infection (Figure S9A). This was accompanied by upregulation of E-box-binding transcription factors, associated with metabolic and circadian rhythm regulation (Figure S9B). We also observed downregulation of genes encoding ribosomal proteins, which, concomitant with an upregulation of genes encoding ubiquitin ligases involved in ER-associated degradation, such as *HRD1*, are indicative of ER stress and induction of the unfolded protein response (Figure S9C, D). Likewise, key effectors of the ER stress response were moderately upregulated in infected cells, including *CHOP* and *HERPUD1*. However, there was limited evidence of coordinated apoptosis induction at the transcriptional level downstream of the ER stress response. We observed modest changes to classical apoptosis markers, accompanied by the expression of anti-apoptotic genes such as *BIRC3* (Fig. S9E). Together, these data suggest that, while infected cells are experiencing translational stress, the response does not commit cells to apoptotic cell death.

Together, our data reveal distinct transcriptional changes in infected and bystander cells. Isolating infected cells based on reporter protein expression enhanced signal-to-noise in transcriptomic analyses and revealed biological pathways that were obscured in bulk populations. Likewise, analysis of the mNG-negative bystander cell population uncovered an early transcriptional response to the inflammatory cytokine milieu, characterized by programmes associated with tissue maintenance and repair.

## Discussion

In addition to its relevance as a human pathogen, HCoV-OC43 serves as an attractive, low-containment model for studying betacoronavirus biology and virus–host interactions. Nevertheless, its use has been constrained by suboptimal growth *in vitro* and a shortage of reliable reagents. Here, we report the development of a toolkit for studying HCoV-OC43, including an optimized cell culture model that supports high-titre viral replication, validated analytical reagents for reverse transcription qPCR and immunoassays and a flexible, plasmid-based reverse genetics platform. These resources are provided in a detailed supplementary handbook.

Our reverse genetics system uses *in vitro* isothermal assembly to generate full-length HCoV-OC43 cDNA from overlapping fragments. This allows rapid genetic manipulation by PCR, followed by a 1 h *in vitro* assembly reaction that is directly transfected into mammalian cells, without the need for an *in vitro* transcription step [87,89], enabling rescue of infectious virus within 4–7 days. This workflow substantially reduces the time required for virus generation compared with yeast-based assembly approaches, in which plasmid assembly and manipulation require ≈14 days, followed by an additional 12–16 days to recover infectious virus [90].

Although a cDNA BAC has been generated for HCoV-OC43 [16], we found that full-length cDNA was unstable and prone to deletion or recombination with bacterial DNA. This may be due to leaky expression of toxic viral gene products in *E. coli*, which hampers plasmid stability and can prevent successful propagation of full-length clones [22,25]. To mitigate this, we included bidirectional transcriptional terminators in our storage plasmid, pCLVR, which allowed HCoV-OC43 genome fragments to be stably propagated and sequenced prior to assembly. Likewise, both *in vitro* ligation and CPER were recently reported for HCoV-OC43 reverse genetics [91,92]. We found that CPER-assembled products were prone to precipitation, compromising rescue efficiency, while *in vitro* ligation requires appropriately positioned restriction sites, which can require the introduction or removal of sites through synonymous mutation [91]. As such, isothermal assembly offers a more flexible and reliable system, which could be developed for other coronavirus species in the future.

Fluorescent reporter coronaviruses often rely on the deletion of an accessory ORF [46,50], at the expense of studying the function of the accessory gene in question. The NS2 gene is dispensable for MHV replication in cell culture [83] and has previously served as a site for reporter gene insertion in HCoV-OC43 reverse genetic systems [32,87, 91, 92]. Consistently, we found mNG was well tolerated at this site, with peak titres comparable to WT and no evidence of reporter deletion after five passages in Mv.1.Lu cells. The mNG reporter was codon optimized to match the low GC content of the HCoV-OC43 genome, to aid insert stability [47]. However, we observed a slight delay in replication and a marginally smaller plaque phenotype, indicative of a small loss of fitness.

We, therefore, developed a fluorescent reporter virus that preserves all endogenous ORFs by insertion of the reporter ORF downstream of a viral TRS to drive expression of a novel subgenomic RNA [54,55, 93,95]. A recently described yeast-based HCoV-OC43 reverse genetics system similarly inserted a fluorescent reporter between the M and N genes, using a minimal TRS core sequence to direct subgenomic RNA transcription [30]. However, gene insertion toward the 3′ end can alter TRS usage and skew sgRNA expression profiles, reducing viral fitness [52,54]. Consistently, Duguay *et al*. reported attenuation and altered transcriptional dynamics in multiple cell types [30].

Across the *Coronaviridae*, accessory genes are frequently acquired between the Spike and Envelope genes [96,100], indicating that this locus may be broadly tolerant to gene insertion. We engineered our reporter, designated ORF4.5, between Spike and ORF5. Transcription was driven by a copy of the M gene TRS. Since coronavirus transcription efficiency is influenced by the RNA sequence context and local structures [51,95, 101,103], we preserved the surrounding sequence around both the inserted TRS and the downstream ORF5 TRS to promote native transcriptional activity [102,103].

Our transcriptomic analysis demonstrated that expression of endogenous sgRNAs in the ORF4.5 reporter virus remained comparable to WT. The ORF4.5 insertion was also well tolerated, with little evidence of attenuation or deletion after serial passage, suggesting that the positioning of the reporter gene and preservation of native TRS flanking sequence elements are important for maintaining optimal viral fitness. We propose that this approach to reporter gene insertion may constitute a broadly applicable strategy for coronavirus engineering, provided that insertion site selection and preservation of native TRS context are carefully considered and optimized.

We then used these reporter viruses to investigate host responses to HCoV-OC43 infection in a human lung cell line, A549. In bulk cell populations, we observed induction of ER stress response genes, a common feature of coronavirus infection [104,106], reflecting both the considerable translational stress during infection and the extensive remodelling of the ER during the establishment of viral replication–transcription complexes [107].

We also observed upregulation of proinflammatory cytokines but limited induction of canonical type I IFN response genes. Comparison of our recombinant viruses, with and without the NS2 protein, indicated that NS2 does not broadly alter innate immune signalling, consistent with its described role as a specific antagonist of RNaseL activation [82,108,110]. This inflammatory response to infection, in favour of type I IFN gene expression, has previously been reported in both A549 and MRC-5 cells infected with HCoV-OC43 [105,111]. A549 cells are known to exhibit altered innate immune signalling compared to primary airway epithelial models [112], and studies of SARS-CoV-2 infection have demonstrated distinct host response profiles in A549, Calu-3 and primary cultures [84,113,116]. Data from primary bronchial cultures suggest that HCoV-OC43 does not induce IFN-*β* secretion or canonical ISG mRNA expression, consistent with our findings [117]. However, this was attributed in part to slow HCoV-OC43 replication kinetics in bronchial cells, and has not been confirmed with transcriptome-wide analyses.

Bulk RNA-seq has limitations for interrogating heterogeneous cell populations, including viral infections, since it samples an average across cells in different infection states [118]. One solution is to perform single-cell RNA sequencing; however, this approach is costly, and sequencing depth may be limited compared to traditional transcriptomic approaches, particularly for low-abundance transcripts [119]. Moreover, most high-throughput single-cell RNA-seq platforms exhibit a strong bias towards sequencing transcript termini [119,120], which would limit the viral transcription analyses we performed here, where detection of sgRNAs relies on chimeric reads spanning leader-TRS-body junctions.

To overcome this, we sorted cells based on reporter fluorescence to enrich for infected cells with active viral translation, as a proxy for productive infection. Compared to our bulk RNA-seq results, we observed stronger upregulation of inflammatory and ER stress pathways and enhanced our resolution of transcriptional reprogramming within infected cells. Expression of genes encoding TNF and IL-1 family cytokines in infected cells correlated with the induction of NF-KB and AP-1 transcription factors, which was accompanied by the induction of transcriptional regulators and histone remodelling factors. Sorting infected cells also revealed a large number of downregulated transcripts, which were masked in bulk populations. These included ribosomal proteins, consistent with an ER stress response, as well as metabolic genes.

Finally, we examined transcriptional responses in bystander cells, which were stringently sorted based on low mNG reporter fluorescence, reflecting an absence of detectable viral translation. Consistently, bystander cells showed minimal induction of ER stress response genes or inflammatory cytokines, signatures of viral infection. However, low levels of viral genomic RNA were detected in this population, suggesting that some bystander cells may have been recently infected or experienced non-productive infections. A previous report, which used cell surface expression of the viral Spike protein to differentiate infected and bystander cells in SARS-CoV-2 infection, similarly observed low levels of viral reads in bystander cells [84]. However, the authors observed that the transcriptional programme of bystander cells closely resembled that of mock-infected cells, with the exception of a small subset of IFN-responsive genes.

By contrast, we observed a transcriptional profile in the mNG-negative bystander cell population, which was distinct from both infected and mock cells. We observed upregulation of genes encoding cell surface receptors involved in cell–cell and extracellular matrix sensing, alongside cytoskeletal components, reminiscent of a wound healing response. Moreover, while proinflammatory cytokine expression was markedly lower than in infected cells, we observed upregulation of cytokine-responsive genes, including NF-KB and AP-1. Notably, the magnitude of transcriptional changes in bystander cells was modest compared to those observed in infected cells. We, therefore, suggest that in these experiments, bystander cells may exhibit a coordinated, low-amplitude response to the local inflammatory environment and tissue perturbation associated with infection.

Together, our engineered reporter virus allowed for sensitive sorting of cells based on active viral translation, facilitating resolution of subtle transcriptional differences in infected and bystander cell populations. Given our reporter virus retains all endogenous ORFs and exhibits little evidence of attenuation, it may be well-suited to more physiologically relevant infection models. While the current study focussed on immortalized cell lines, future experiments in heterogeneous culture systems, such as primary nasal cell cultures, will be informative for resolving cell type-specific susceptibility and host response to HCoV-OC43 infection, while overcoming biases associated with immortalized cell lines. Likewise, combining cell sorting with single-cell transcriptomic approaches could further resolve the bystander cell population to differentiate host responses in cells undergoing abortive infection from bona fide uninfected cells.

Our tools address a critical gap in the experimental tractability of HCoV-OC43, providing a foundation for more detailed molecular and cellular studies of a historically understudied human coronavirus. These open new avenues of research in HCoV-OC43, a relevant human pathogen and a model biosafety level 2 betacoronavirus and set a foundation for the development of new tools for other equally understudied coronaviruses.

## Supplementary material

10.1099/jgv.0.002282 Supplementary Material 1.

## Abbreviations

CPE: cytopathic effect
CPER: circular polymerase extension reaction
DMEM: Dulbecco’s Modified Eagle Medium
dpi: days post-infection
ER: endoplasmic reticulum
GSEA: gene set enrichment analysis
HCoV: human coronavirus
L: linker fragment
M: membrane protein
MHV: mouse hepatitis virus
mNG: mNeonGreen
qPCR: quantitative PCR
RG: reverse genetics
sgRNAs: subgenomic RNAs
TAR: transformation-associated recombination
TRS: transcription regulatory sequence
