# Supplementary material for: A stable subgenomic reporter coronavirus enables transcriptional profiling of bystander cells
Source: J Gen Virol. 2026 Jun 16;107(6):002282. doi: 10.1099/jgv.0.002282 (PMC13271436; doi:10.1099/jgv.0.002282)
Supplement: Supplementary Material 1. [file jgv-107-02282-s001.pdf]

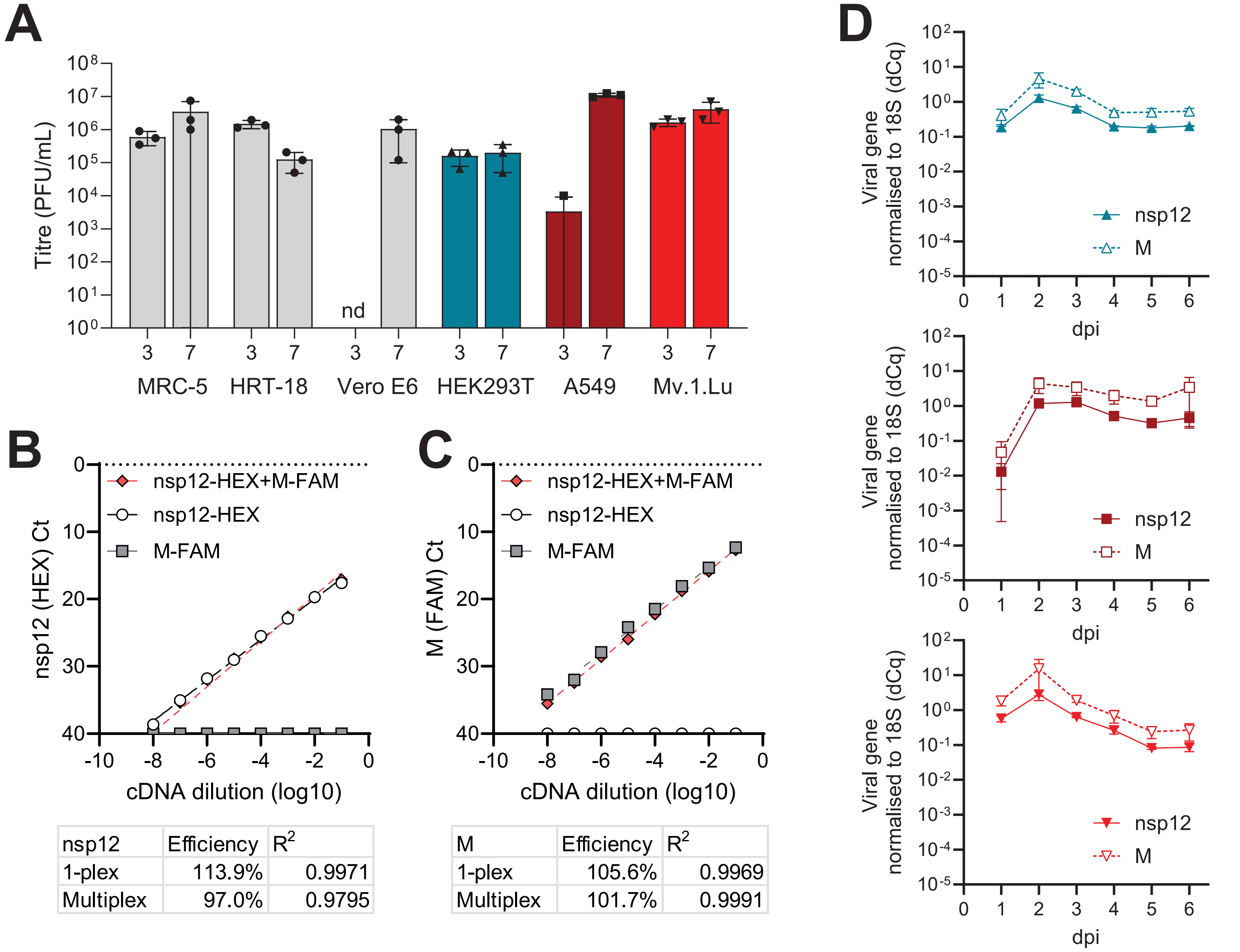

nsp12	Efficiency	R^2
1-plex	113.9%	0.9971
Multiplex	97.0%	0.9795

M	Efficiency	R^2
1-plex	105.6%	0.9969
Multiplex	101.7%	0.9991

Figure S1. Growth and analysis of HCoV-OC43 in cell culture. **A.** Infectious titres of HCoV-OC43 released from the indicated cell lines at 3- and 7-days post infection (dpi) in plaque forming units (PFU)/mL. Data are means and standard deviations of three biological replicates. nd, not detected. **B-C.** Standard curves of a HEX-labelled nsp12 primer-probe set (B) and FAM-labelled M gene primer-probe set (C), either alone (white, grey) or multiplexed (red), using gene-specific PCR amplicons, amplified from cDNA from HCoV-OC43-infected cells, as templates, to test for specificity. Primer efficiency ($E = -1 + 10^{(-1/Slope)}$) and R^2 values are shown. **D.** Growth of HCoV-OC43 in 293T (upper), A549 (middle) or Mv.1.Lu (lower) cells infected at an MOI of 0.05, analysed by RT-qPCR on RNA from cell lysates. Viral gene expression (nsp12, solid line and M, dashed line) was normalised to host 18S RNA (ΔCq). dpi, days post infection. Data are means and standard deviations of at least three biological replicates.

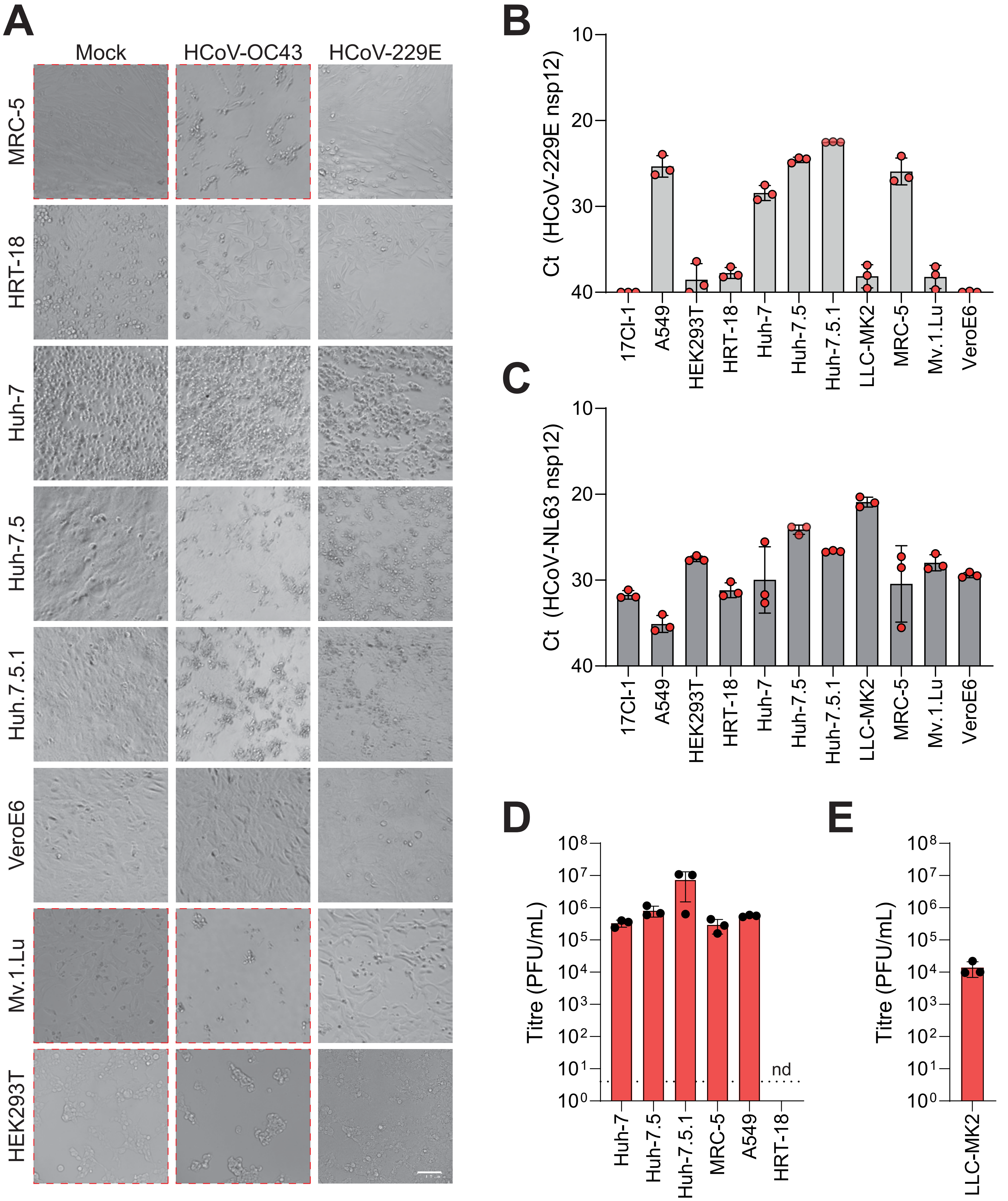

Figure S2. Cell culture conditions for human alphacoronaviruses. **A.** Light microscopy showing eight cell lines infected with HCoV-229E, or mock infected, at an MOI of 0.0001, five days post infection. HCoV-OC43 is included for comparison and dashed boxes indicate panels which also appear in Figure 1C and are included here for reference. Scale bar represents 100 μm. **B-C.** RT-qPCR analysis of RNA from supernatants from HCoV-229E (B) or HCoV-NL63 (C) -infected cells, five days post infection. Data represent means and standard deviations of three biological replicates. **D-E.** Plaque assay of supernatants from B and C, respectively. Plaque assays for HCoV-229E (D) were performed in Huh-7 cells and for HCoV-NL63 (E) in LLC-MK2 cells. nd, not detected.

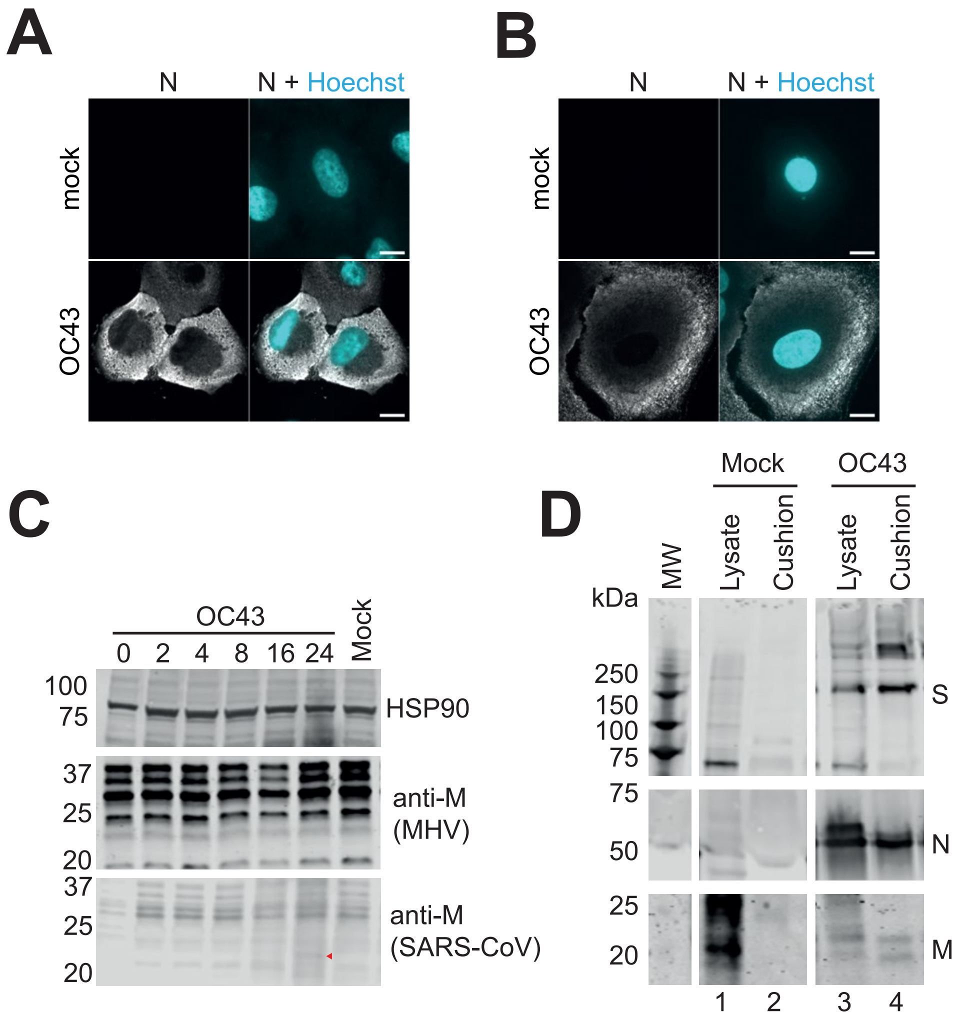

Figure S3. Antibody validation. **A-B.** Immunofluorescence microscopy of HCoV-OC43-infected A549 cells using two different anti-N antibodies: a rabbit polyclonal (A, SinoBiological 40643-T62, 1:1000) and a mouse monoclonal (B, Merck MAB9013, 1:1000). Scale bars represent 10 µm. **C.** Western blot analysis of Mv.1.Lu cells infected with HCoV-OC43 at an MOI of 10 for up to 24 hours, probed with two anti-M antibodies, a mouse monoclonal raised against MHV and a rabbit polyclonal raised against SARS-CoV. Weak M detection at 24 hpi is indicated by a red arrowhead. **D.** Western blot of viral supernatants concentrated by ultracentrifugation through a 30% sucrose cushion, compared to cell lysates. Background signal is higher in mock-infected cell lysates (lane 1), due to substantial cytopathic effect in infected cells.

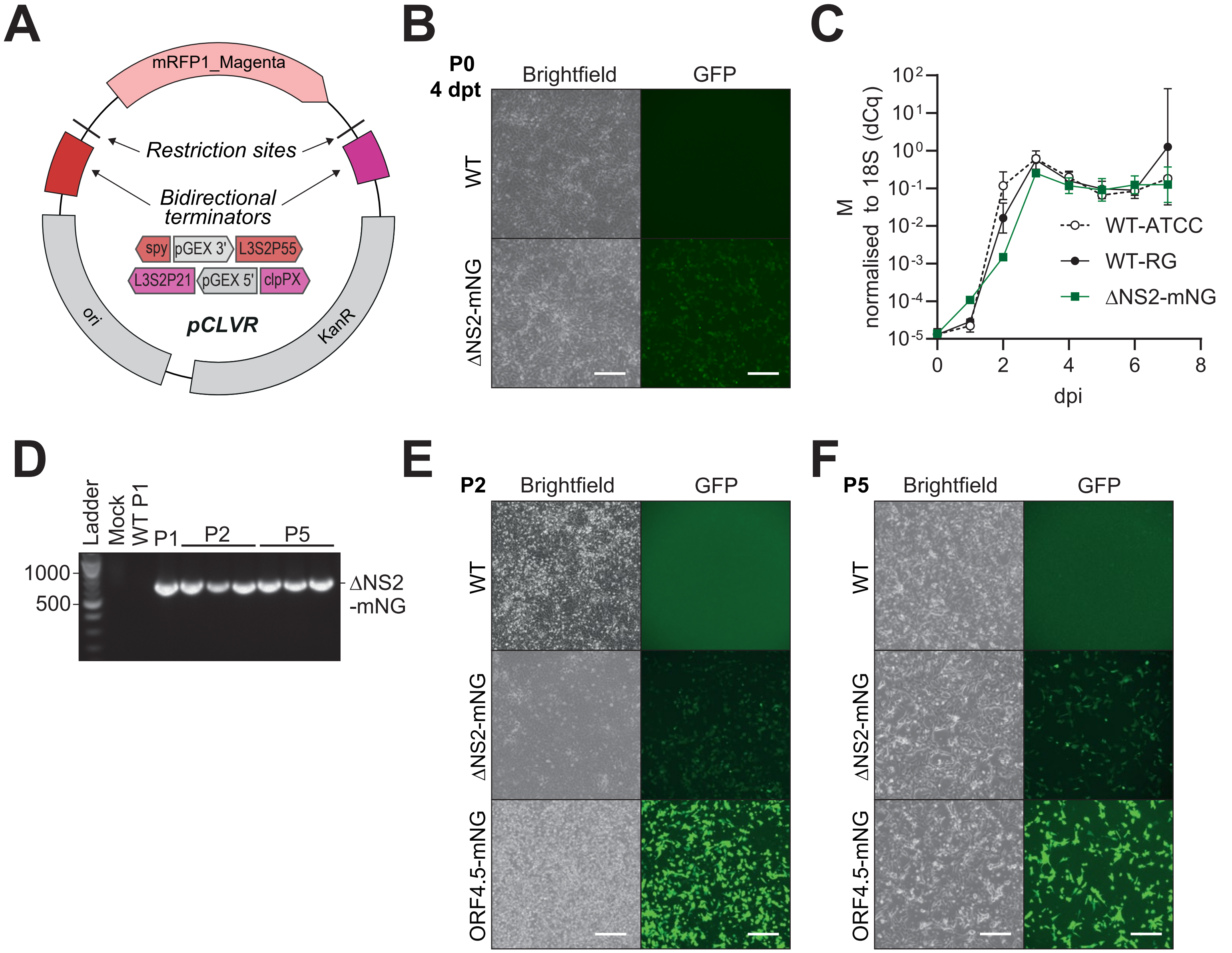

Figure S4. Rescue and passage of reverse genetics-derived HCoV-OC43. **A.** Schematic of the pCLVR storage vector, showing restriction sites flanking an mRFP1_Magenta chromoprotein, which is excised upon insert cloning. Upstream (red) and downstream (pink) bidirectional transcriptional terminators, including pGEX primer binding sites for plasmid sequencing, as well as bacterial replication (ori) and antibiotic resistance (KanR) elements, are indicated. **B.** Brightfield and fluorescence microscopy analysis of HEK293T cells transfected with *in vitro* assembled HCoV-OC43 infectious clones, four days post transfection. **C.** Replication of WT (ATCC isolate, dashed line) and RG-derived HCoV-OC43 in Mv.1.Lu cells (MOI 0.01) measured by RT-qPCR on RNA from cell lysates, against the viral M gene normalised to host 18S rRNA (ΔCq). Data are means and standard deviations of three biological replicates. **D.** Agarose gel analysis of PCR amplicons within the ΔNS2-mNG insertion site, amplified from RNA purified from supernatants, from passage 1 (P1), P2 or P5. Three biological replicates of P2 and P5 were analysed. **E-F.** Brightfield and fluorescence microscopy analysis of Mv.1.Lu cells infected with WT and fluorescent reporter viruses at passage 2 (P2, C) and P5 (D). Scale bars represent 200 μm.

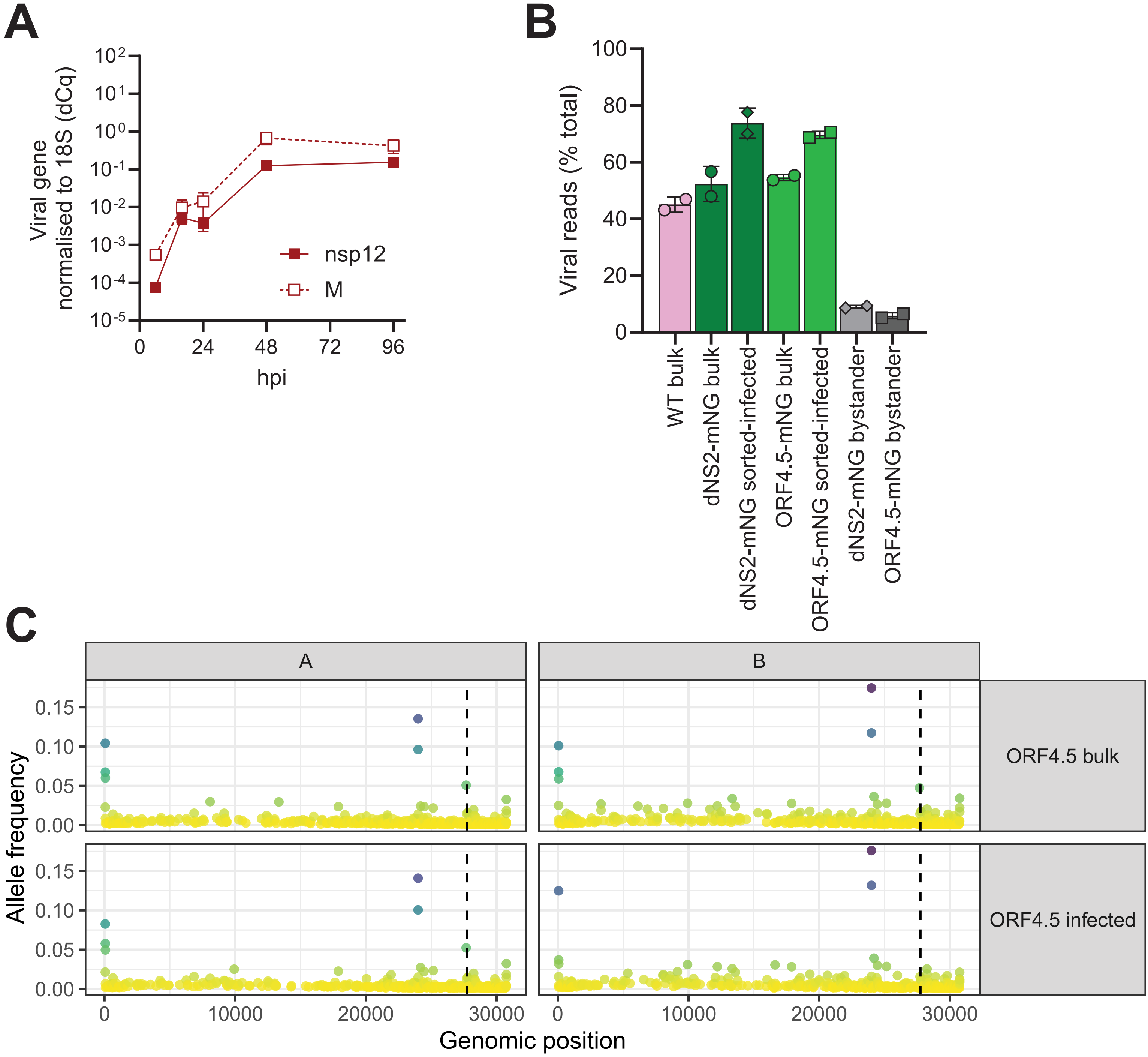

Figure S5. Transcriptomic analysis of HCoV-OC43-infected A549 cells. **A.** Growth of HCoV-OC43 in A549 cells (MOI 1), analysed by RT-qPCR on RNA from cell lysates. Viral gene expression (nsp12, solid line and M, dashed line) was normalised to host 18S RNA (ΔCq). dpi, days post infection. Data are means and standard deviations of three biological replicates. **B.** Proportion of viral reads in infected A549 cells, from bulk and sorted cell populations. Data represent means and standard deviations of two independent experiments. **C.** Frequency of single nucleotide polymorphisms in HCoV-OC43-ORF4.5-mNG transcriptomic data, determined by lofreq, mapped to WT coordinates. The ORF4.5 insertion site is indicated by a dashed line.

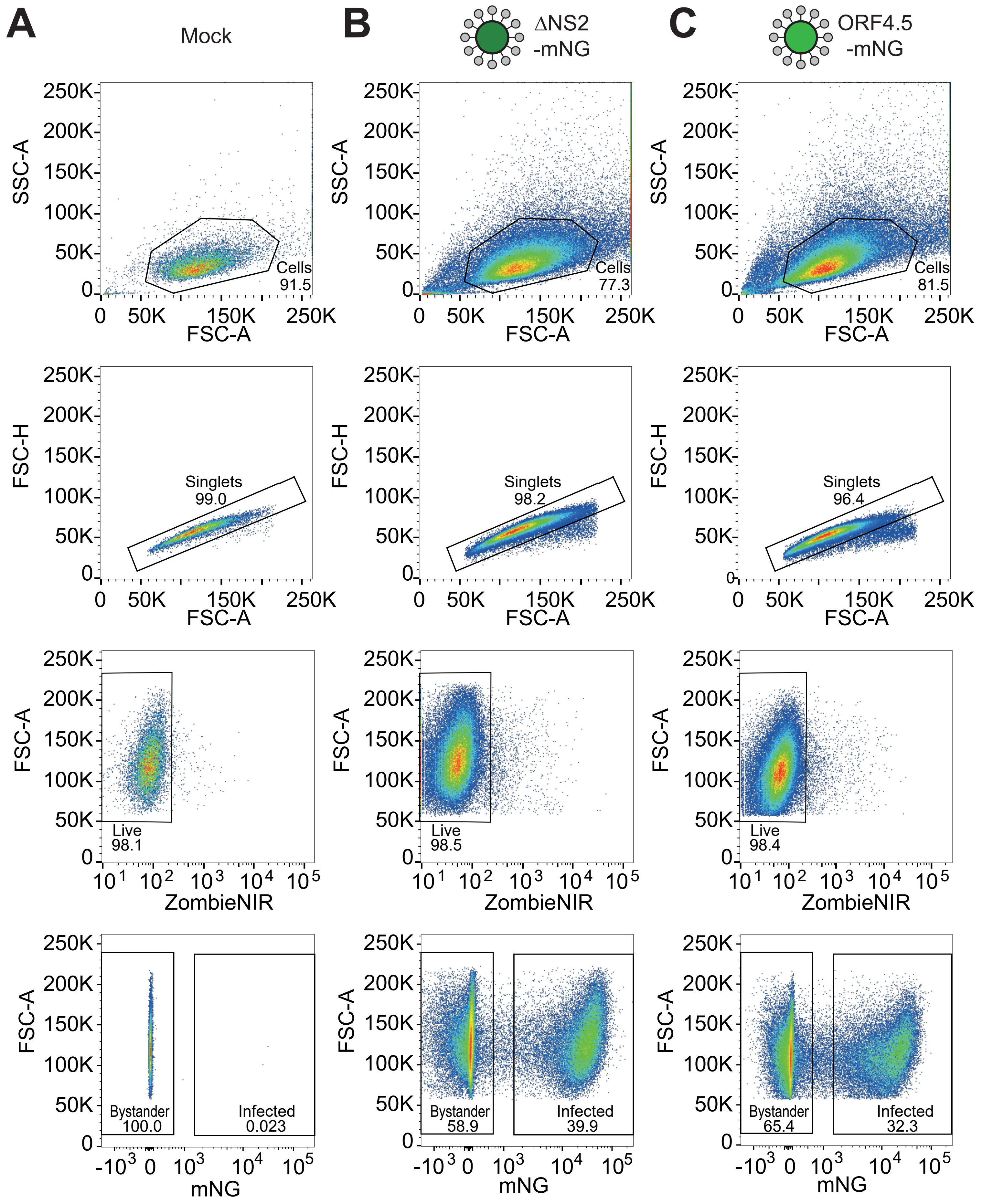

Figure S6. Sorting of cells infected with HCoV-OC43 reporter viruses. **A-C.** Gating strategy for isolation of live singlets and separation of infected (mNG-positive) and bystander (mNG-negative cells), for mock infected (A), HCoV-ΔNS2-mNG-infected (B) or HCoV-OC43-ORF4.5-mNG-infected (C) A549 cells, MOI 1, 24 hours post infection.

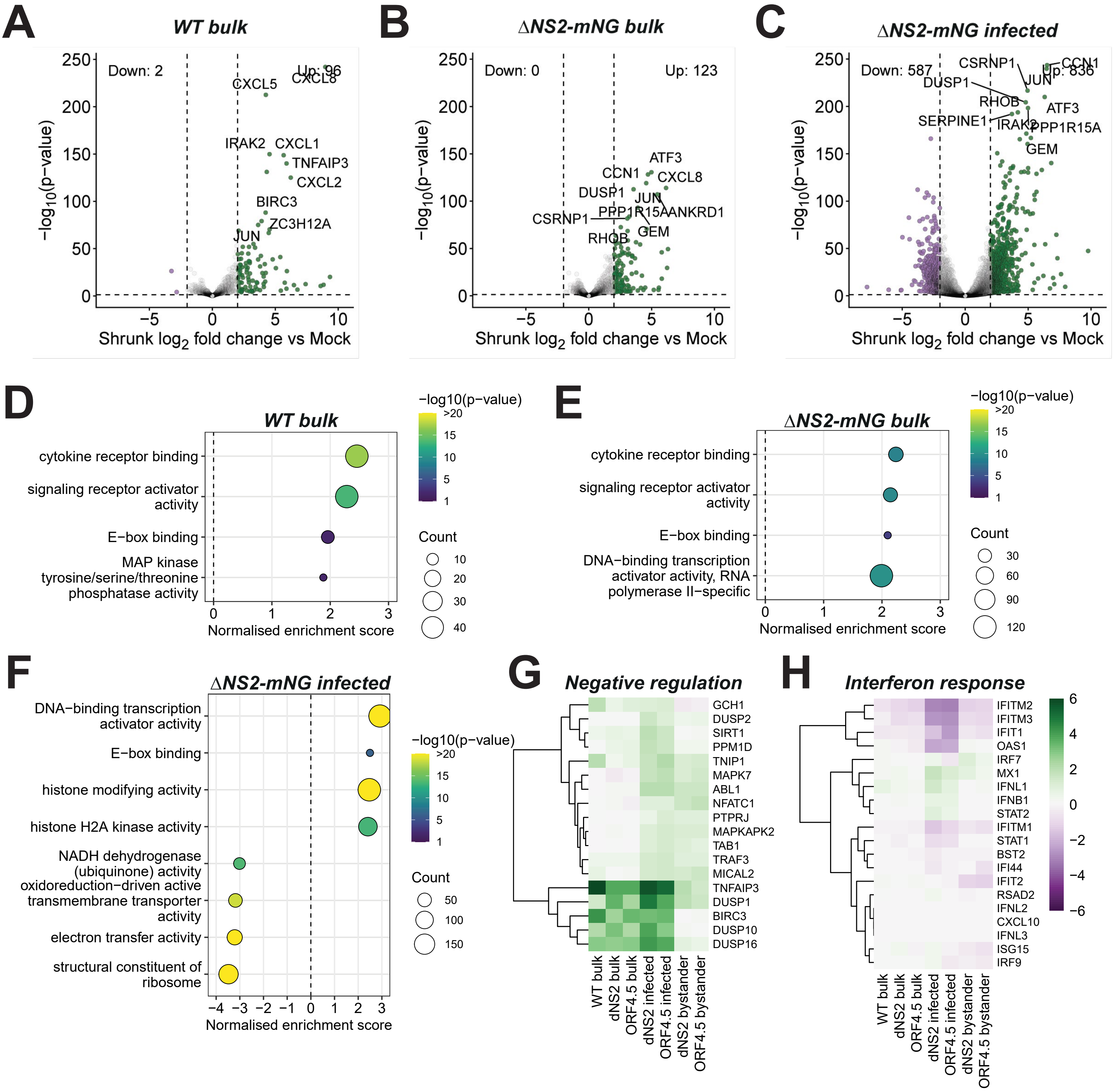

Figure S7. Host transcriptional responses in HCoV-OC43-WT and HCoV-OC43-ΔNS2-mNG infection. **A-C.** Volcano plots showing differentially expressed genes in HCoV-OC43-WT infection (A), or bulk (B) or sorted (C) HCoV-OC43-ΔNS2-infected cells, expressed as shrunk $\log_2$-fold change over mock infected cells. Significantly up- or downregulated genes (shrunk $\log_2$ fold change >±2, p value <0.05) are coloured and top 10 most significant genes are labelled. **D-F.** Gene set enrichment analysis of differentially expressed genes, based on molecular function, in HCoV-OC43-WT infection (D), or bulk (E) or sorted (F) HCoV-OC43-ΔNS2-infected cells. Functions are ranked by normalised enrichment score and coloured by significance. **G-H.** Heatmaps showing the expression of genes encoding negative regulators of NF-kappaB and MAPK signalling (G) and expression of classical type I IFN response genes (H), coloured by shrunk $\log_2$ fold change (shFC) in expression compared to mock cells.

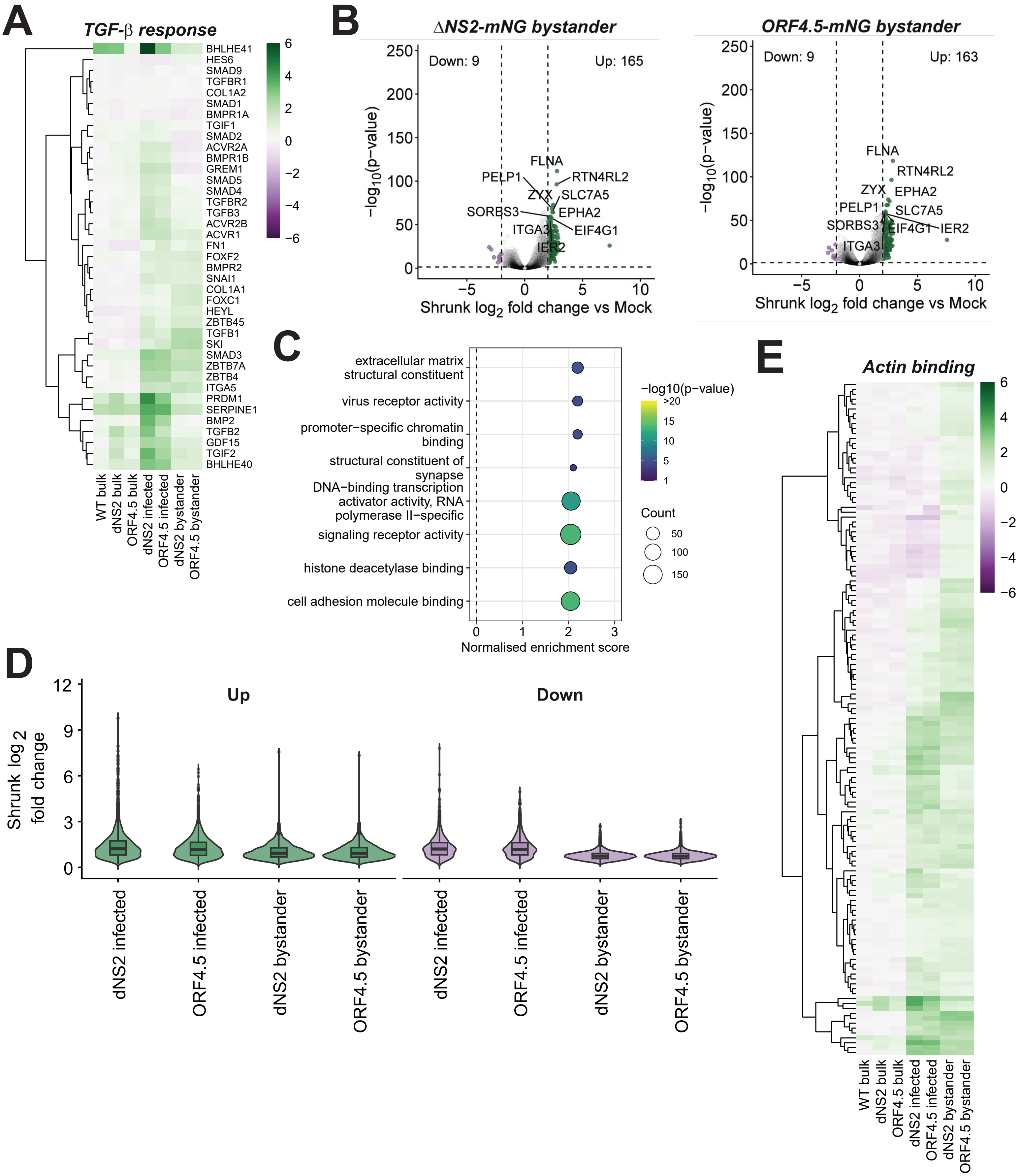

Figure S8. Actin filament induction in bystander cells. **A.** Heatmap showing the expression of genes involved in TGF-beta signalling, coloured by shrunk log_2 fold change (shFC) in expression compared to mock cells. **B.** Volcano plots showing differentially expressed genes in bystander (mNG-negative) cells following HCoV-OC43-ΔNS2-mNG (left) or HCoV-OC43-ORF4.5-mNG (right) infection, expressed as shrunk log_2-fold change over mock-infected cells. Significantly up- or downregulated genes (shrunk log_2 fold change $>\pm 2$, p value <0.05) are coloured and top 10 most significant genes are labelled. **C.** Gene set enrichment analysis of differentially expressed genes in ΔNS2-mNG bystander cells, based on molecular function. Functions are ranked by normalised enrichment score and coloured by significance. **D.** Summary of up- (green) and down- (purple) regulated gene expression in infected and bystander cells, shown as shrunk log_2-fold change over mock-infected cells, for all genes where $p<0.05$. **E.** Heatmap showing the expression of genes related to actin binding, coloured by shrunk log_2 fold change (shFC) over mock-infected cells.

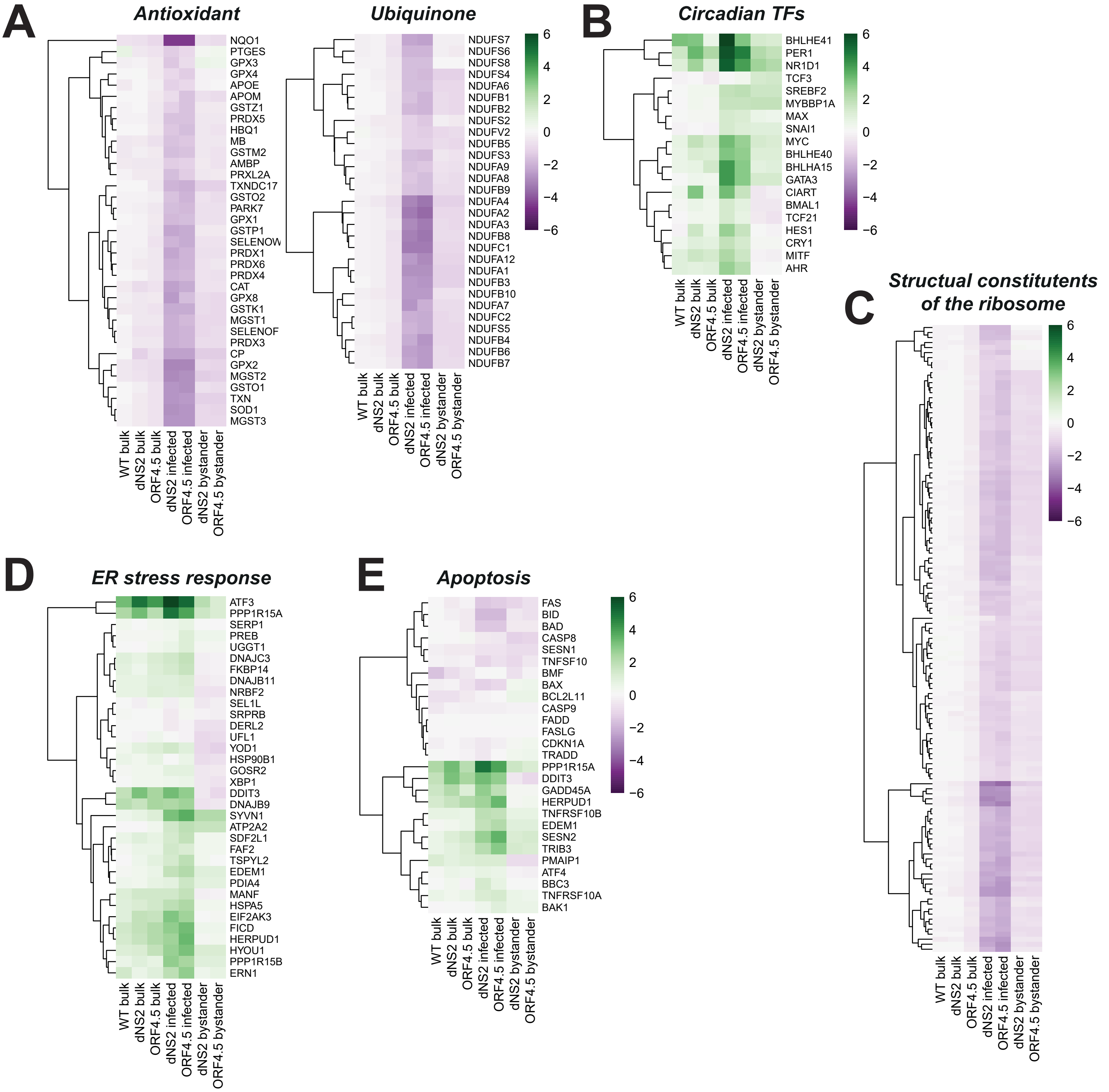

Figure S9. Downregulation of metabolism and induction of the ER stress response in infected cells. **A-E.** Heatmaps showing the expression of genes involved in oxidative phosphorylation (A), transcription factors associated with circadian rhythm regulation (B), structural constituents of the ribosome (C), the endoplasmic reticulum stress response (D) and pro-apoptotic markers (E), coloured by shrunk $\log_2$ fold change (shFC) over mock-infected cells. N.b. some ER stress response genes (D) are also represented in the apoptosis heatmap (E), since these pathways are interlinked.

Supplementary protocol: reagents and methods for the propagation and analysis of HCoV-OC43

Ciaran Gilbride[1,2#], Joe Hemsley-Taylor[1], Catarina Nunes[1], Rebecca Penn[1], James Boot[1,3], Nima Pieris[4], Rupa Tripathy[1], Ziyi Yang[1], Matthew Hutchinson[1], Olivia K Platt[1], Rachel Ulferts[5], Richard Mitter[3], Molly Strom[2], Nuno B. Santos[1], David L.V. Bauer[1], Harriet V. Mears[1,4]

[1] RNA Virus Replication Laboratory, The Francis Crick Institute, London, United Kingdom
[2] Viral Vector Core, The Francis Crick Institute, London, United Kingdom
[3] Bioinformatics and Biostatistics STP, The Francis Crick Institute, London, United Kingdom
[4] School of Biochemistry & Biomedical Sciences, University of Bristol, Bristol, United Kingdom
[5] Cell Biology of Infection Laboratory, The Francis Crick Institute, London, United Kingdom
[#] Current address: Faculty of Engineering, Imperial College London, London, United Kingdom

Contents

Biosafety and HCoV-OC43 2
Culture and quantification of HCoV-OC43 3
HCoV-OC43 infection 3
HCoV-OC43 propagation 3
HCoV-OC43 plaque assay 3
RT-qPCR 4
Primer-probe sequences 4
Antibodies 4
Reverse genetics components & plasmids 5
Materials 5
Plasmids 5
Amplification primers 6
Reverse Genetics protocol 7
Generation of fragments by PCR 7
Assembly by HiFi 7
Transfection 8
Cloning archival fragments in pCLVR plasmids and propagation in *E. coli* 9
Linearisation primers 9
Archiving Primers 9
Preparation of fragments 9
Transformation 10
Amplicon sequencing of HCoV-OC43 11
Reagents: 11
Sequencing primers 11
Protocol 11

Biosafety and HCoV-OC43

⚠️ This document is NOT a substitute for complete risk assessments and protocols, and no work should commence until you have read these risk assessments, local rules and relevant Codes of Practice. You must also have received appropriate training, and been cleared as fit to undertake this work by your organisation's Occupational Health provider. The authors of this document and The Francis Crick Institute assume no liability for any work using the materials and methods described here.

HCoV-OC43 strains can cause mild respiratory disease in healthy adults and children. In the UK, the Advisory Committee on Dangerous Pathogens (ACDP) has classified HCoV-OC43 as a Hazard Group 2 pathogen, allowing its use in Containment Level 2 laboratories; in most other countries, it is classified similarly and can be used at the equivalent of a BSL-2 laboratory.

As an enveloped virus, like SARS-CoV-2 and influenza viruses, HCoV-OC43 is readily inactivated by ethanol, phenol, heat, UV irradiation, chemical fixation, and many detergents. Validated disinfectants and inactivation protocols used for SARS-CoV-2 are likely effective for HCoV-OC43, but attention must be paid to the higher titres routinely achieved in culture for HCoV-OC43.

The strain used here as a model was obtained from ATCC (VR-1558) and is highly lab-adapted to tissue culture. We have confirmed that there is durable, widespread immunity to this strain in the population: using a high-throughput live virus microneutralisation assay and serum samples from a cohort of 282 healthy UK adults enrolled in the Crick/UCLH Legacy study (clinical trials registration NCT04750356), we found that all participants were able to neutralise HCoV-OC43. This immunity did not wane over a 12-month period, implying this immunity is lifelong: neutralisation titres in the cohort consistently had a median IC_{50} of ~350 (Carr et al., bioRxiv, 2023), which is above the correlate of protection for most respiratory viruses.

In the UK, you must not carry out genetic modification of HCoV-OC43 unless you have notified the UK Health and Safety Executive and received their consent. It is essential that you comply with the terms of your specific GM license and local rules.

At The Francis Crick Institute, our license from the HSE permits us to make genetic modifications for the following purposes only:

- ☐ 1 - Insertion of tags to monitor infection
- ☐ 2a - Deletion of viral genes, coding regions, ORFs, or RNA motifs
- ☐ 2b - Creation of attenuating / truncating / catalytic mutations
- ☐ 3 - Re-generation of viruses that have been sequenced previously
- ☐ 4 - Introduction of naturally-occurring mutations into the background of well-characterised laboratory strains
- ☐ 5 - Adaptation to animal models

Under no circumstances are we allowed to introduce mutations that would generate resistance to available antivirals or antibody treatments, nor are we allowed to introduce any mutations that would be considered "gain of function" (see European Academies' Science Advisory Council Policy Report 27).

When working, we also must maintain physical and temporal separation of HCoV-OC43 from other coronaviruses as a precaution against spurious recombination arising from accidental coinfection.

Culture and quantification of HCoV-OC43

HCoV-OC43 infection

- HCoV-OC43 readily infects Mv.1.Lu (ATCC CCL64) cells, HEK293T cells and A549 cells.
- Cells can be infected either in suspension, or on monolayers at 50-80% confluency.
- Infections are performed in DMEM supplemented with 2% foetal calf serum (FCS).
    1. Infect cells at the desired multiplicity of infection (MOI) in a low volume of media (e.g. 0.25 mL in a 12-well plate), with occasional agitation or on a rocker for 30-60 minutes.
    2. Remove inoculum and replace with fresh 2% FCS DMEM.
    3. Incubate at 33 °C, 5% CO_2 for the desired length of time.

HCoV-OC43 propagation

In Mv.1.Lu or HEK293T cells. Adapted from Coronaviruses: Propagation, Quantification, Storage, and Construction of Recombinant Mouse Hepatitis Virus - PMC

1. Seed T175 flasks, aiming for 50-70% confluency on the day of infection.
2. For infection, dilute OC43 to an MOI of 0.0001 PFU/cell in 5 mL 2% FCS DMEM.
3. Remove media from cells and add 5 mL of OC43 inoculum.
4. Incubate at room temperature, rocking for 1 hour.
5. Remove inoculum and replace with 20-25 mL 2% FCS DMEM.
6. Incubate at 33 °C 5% CO_2 for 3-5 days until CPE is clearly visible and cells have started to detach.
    a. Optionally: freeze the entire flask at -80 °C for at least 1 hour to ensure complete cell lysis.
    b. Thaw flask at room temperature or in a water/bead bath.
7. Clarify supernatant by centrifugation at >3,000 x *g* for 10 minutes at 4 °C.
8. Aliquot virus and store at -80 °C.

HCoV-OC43 plaque assay

In Mv.1.Lu or A549 cells. Adapted from Improved plaque assay for human coronaviruses 229E and OC43 - PMC (nih.gov)

1. Seed cells for 95-100% confluency on the day of infection.
    a. e.g. one day prior to infection, seed wells in 12-well plates at $4x10^5$ Mv.1.Lu cells per well in 1ml 10% FCS DMEM. Incubate at 37 °C 5% CO_2 for 24 hours.
2. Prepare serial dilutions of OC43 in 2% FCS DMEM.
    a. e.g. dilute 30 µL into 270 µL media, from 10^{-2} to 10^{-7}
3. Remove media from cells and infect with 250 uL of diluted inoculum.
4. Incubate at room temperature for 1 hour, with occasional agitation or continual slow rocking.
5. Prepare Avicel overlay:

	Final concentration	mL for 100 mL
2X MEM	1X	50
FCS	10 %	10
Pen/strep (optional)	1 %	1
2.4 % Avicel (in water)	0.94 %	39

6. Without removing inoculum, add 1 mL of Avicel overlay to each well and incubate for 4-5 days at 33 °C 5% CO_2.
    a. Not removing the inoculum avoids cells drying out.
    b. This dilutes Avicel to 0.75%.
7. Remove overlay and fix cells with 2 mL 4% paraformaldehyde (PFA) in PBS for 30-60 minutes.
8. Remove PFA and add ~1 mL of 0.2% w/v Toluidine Blue in PBS and incubate for >60 minutes.
9. Gently rinse plates with water to remove staining solution.
10. Count plaques and calculate viral titre.

RT-qPCR

Primer-probe sequences

M primer-probes are from [Development of One-Step, Real-Time, Quantitative Reverse Transcriptase PCR Assays for Absolute Quantitation of Human Coronaviruses OC43 and 229E - PMC (nih.gov)]

The nsp12 primer-probes were designed with similar properties to the M set to allow multiplexing. This has been validated for two-step RT-qPCR only using the Taqman multiplex master mix (TaqMan™ Multiplex Master Mix 1 mL | Buy Online | Applied Biosystems™ | thermofisher.com) on a Quantstudio3 using the default Taqman run method. If using another kit, check linear amplification with a cDNA standard curve.

Target	Forward	Probe	Reverse
M	5’-ATGTTAGGCCGATAATTGAGGACTAT-3’	5’-HEX-CATACTCTGACGGTCACAAT-BHQ-3’	5’-AATGTAAAGATGGCCGCGTATT-3’
nsp12	5’-ACGTGGTGTTCCTGTAGTTATAGG-3’	5’-FAM-CAGCCACCATAAAATTTAGTGGT-BHQ-3’	5’-GGCGGCGTAACATATCATCC-3’

20X primer/probe mixes (in water):

e.g. for 10 µL	Forward (100 µM stock)	Probe (10 µM stock)	Reverse (100 µM stock)
M	0.6 µL (6 µM, 300 nM final)	4 µL (4 µM, 200 nM final)	0.6 µL
nsp12	0.6 µL	5 µL (5 µM, 250 nM final)	0.6 µL

For absolute quantification, make cDNA standards from sequencing amplicons 5 (nsp12) and 9 (M). Gel extract amplicons and quantify accurately (e.g. by Qubit) and dilute down carefully.

Antibodies

Target	Supplier	Cat. No.	Species (clonality)	Western blot	Immuno-fluorescence	Immuno-precipitation
S	The Native Antigen Company	PAB21478-100	Rabbit polyclonal	1:1000	Background mitochondrial signal	Not tested
S	Sino Biological	68086-MM29	Mouse monoclonal (clone 29)	Not tested	1:1000	Not tested
N	Sino Biological	40643-T62	Rabbit polyclonal	1:1000	1:1000	Not tested
N	MRC-PPU (Rihn et al. 2021 PLoS Biol)	N/A	Sheep polyclonal	1:1000	1:1000	10 µg/100 µL dynabeads
N	Merck	MAB9013	Mouse monoclonal (542-7D)	1:1000	1:1000	Not tested
M	Antibodies online	ABIN1887462	Rabbit polyclonal	1:200 High background unless from purified virions	1:200 (background nuclear signal)	10 µg/100 µL dynabeads High background in mock cell lysates

Reverse genetics components & plasmids

Materials

- Nuclease-Free water
- Agarose
- LB broth
- LB agar
- Kanamycin
- NEB Stable competent E.coli (New England Biolabs, C3040I)
- SuperFi II 2x master mix (Invitrogen, 12368010)
- Nucleospin Gel and PCR Clean up (Macherey-Nagel, 740609.250)
- Hifi DNA Assembly MasterMix (New England Biolabs, E2621L)
- Primestar GxI polymerase (Takara Biosciences, R050A)
- Lipofectamine 3000 transfection reagent (Invitrogen, L3000001)
- Opti-MEM reduced serum medium (Gibco, 11058021)
- Dulbecco's modified eagle medium
- Foetal Bovine Serum
- Penicillin/Streptomycin

Plasmids

Plasmid name	Plasmid size	Fragment size	Primers
OC_UTR_Linker	3118bp	1473bp	OC5PrimeRev+OC3primeFw
OC1_B1_pCLVR	5030bp	3000bp	OC1Fw+OC1Rev
OC2_B1_pCLVR	5032bp	3002bp	OC2Fw+OC2Rev
OC3_B1_pCLVR	5029bp	2999bp	OC3Fw+OC3Rev
OC2/3_B1_pCLVR	7960bp	5930bp	OC2Fw+OC3Rev
OC4_A1_pCLVR	5007bp	3000bp	OC4Fw+OC4Rev
OC4/5_A1_pCLVR	7937bp	3002bp/5930bp	OC5Fw/OC4Fw+OC5Rev
OC6_A1_pCLVR	5010bp	3003bp	OC6Fw+OC6Rev
OC7_B1_pCLVR	5033bp	3000bp	OC7Fw+OC7Fw
OC6/7_A1_pCLVR	7940bp	5933bp	OC6Fw+OC7Rev
OC8_B1_pCLVR	5036bp	3003bp	OC8Fw+OC8Rev
OC8mNG_B1_pCLVR	5051bp	3018bp	OC8Fw+OC8Rev
OC9_B1_pCLVR	5029bp	2999bp	OC9Fw+OC9Rev
OC8/9_B1_pCLVR	7965bp	5932bp	OC8Fw+OC9Rev
OC9/10_B1_pCLVR	7959bp	5929bp	OC9Fw+OC10Rev
OC10_B1_pCLVR	5030bp	3000bp	OC10Fw+OC10Rev
OC10/11_B1_pCLVR	6409bp	4379bp	OC10Fw+OC11Rev
OC11_B1_pCLVR	3479bp	1449bp	OC11Fw+OC11Rev
OC_UTR_BAC	8695bp	8656bp	OC5PrimeRev+OC3primeFw

Amplification primers

Primer name	Primer Sequence
OCUTR5PrimeRev	GTTTAGATTACGAAAAGATCTAACAAGAGATCAGTGAAAC
OCUTR3PrimeFw	CAGCATGTTAAGTTACCACCCAGTAATTAGTAAATG
OC1Fw	ATTGTGAGCGATTTGCGTGCGT
OC1Rev	TAACTTTAGCACCTACACCTTCAAGCTCC
OC2Fw	GTGGTGATTGATGCCATAGAAGAGAAACTTTCT
OC2Rev	GCTTTAATAATACGCTGAGTATAATACTTACCTCCGTCA
OC3Fw	GAGTATAAACCTGACTTGTCACAATATTATTGTGACGG
OC3Rev	GGCGTATAACACTGCACTCCATCAG
OC4Fw	GATATGGTTATCATGTGTTGCACTTTATTACACATGC
OC4Rev	TAGAAGCAACATGCAAATGTTGCAAGCA
OC5Fw	CAAAATTGACTGATGTCAAATGTGCTAATGTCGTC
OC5Rev	AAACAAACAACAACTGCTTAATGTCCACCATG
OC6Fw	GCTATTACTGATTATAATTATTATAAGTATAATTTGCCCACCATG
OC6Rev	CTGACTATTATATGGGCTAATAAAAACAGCTTTATGCCAC
OC7Fw	GATTAATAAGTTTTTGAAGGCTAACCCTTTGTGGC
OC7Rev	AAAGTCATAACTTTCTCATCGTTACACCAAAGCATAA
OC8Fw	AGTTAATGTTAATGTTGATTTTAAAGATTTTCAGTTTATGCTTTGG
OC8Rev	CACACAAATGGATGAATCAGGGATATTAATATCAGCAG
OC9Fw	GCCTCTCTACCCCTATGGCAGATG
OC9Rev	AAACCGACATCAGATAACTTTACTTTATCAAAAAGTAAATCCTC
OC10Fw	CTTATGCTTCCAGTAGATCTGCTATAGAGGATTTACTTT
OC10Rev	CACCTTGTCCTTCTACAAACTCAAACTCCTTTC
OC11Fw	TATTCTTGGTTCTCTGGAATTACTCAGTTTCAAAAGG
OC11Rev	GTGATTCTTCCAATTGGCCATAATTAACTT

Reverse Genetics protocol

The reverse genetics protocol for HCoV-OC43 contains three parts: generation of fragments by PCR, *in vitro* assembly of genome fragments, and finally rescue of virus by transfection of assembled DNA.

Generation of fragments by PCR

1. For each OC43 fragment, use a single template and pair of primers. Plasmid and primer combinations are given in the plasmids table.
2. Assemble PCR reactions using a high-fidelity polymerase, e.g. SuperFi II:

Reagent	Volume
Plasmid Template (10-50 ng)	~1 µL
Fw and Rev primer (100 µM)	0.3 µL each
Nuclease-free water	Up to 25 µL
SuperFi II 2x Master Mix	25 µL

3. Run the following protocol in a thermocycler to generate PCR fragments:

Stage	Temperature	Time
1. Initial Denaturation	98ºC	3 minutes
2. Denaturation	98ºC	10 seconds
3. Annealing	60ºC	10 seconds
4. Extension	72ºC	30 seconds per kb (see below)
5. Final extension	72ºC	5 minutes

12-24x cycles 2-4

Fragment	Extension time
Single	1 min 30 s
Double	3 min
OC10/11	2 min
Linker	45 s

4. Run 3-5 µL of the reaction on a 1% agarose gel to determine successful PCR. A single band should be observed for each reaction.
5. Purify PCR products (e.g. using Nucleospin Gel and PCR clean up kit) and elute in 20 µL nuclease-free water.
6. Store fragments at -20 °C.

Assembly by HiFi

1. Into a fresh 0.2 mL PCR tube, add:
    a. 100 ng of each 3 kb fragment <u>or</u> 200 ng of each 6 kb fragment
    b. 150 ng of fragment OC10/11 and 50 ng linker, or 100 ng of fragment OC11/Linker
2. Add an equivalent volume of NEB HiFi 2x master mix to the tube and mix by pipetting gently.
3. Incubate at 50 °C for 1 hour, followed by heat inactivation at 65 °C for 15 minutes.
4. Assembled products can be used immediately or stored at -20 °C until transfection.

Transfection

Day 0

1. Seed HEK293T cells for ~70 confluency on the day of transfection.
    a. e.g. in a 6-well plate, seed $5x10^5$ HEK293T cells per well.

Day 1

2. Add the entire assembly reaction to 100 µL Opti-MEM.
3. For each transfection, prepare 100 µL of Opti-MEM and add 2 µL P3000 and 3 µL Lipofectamine 3000.
4. Mix the DNA and transfection reagents and incubate at room temperature for 20 minutes.
5. Remove media from HEK293T cells and add 1.8 mL 2% FCS DMEM.
6. Add DNA/lipofectamine mixture dropwise to each well with gentle agitation.
7. Incubate overnight at 33 °C 5% CO_2.
8. Exchange media for 2 mL fresh 2% FCS DMEM.
9. Incubate at 33 °C 5% CO_2 for 4-7 days.
    a. Fluorescent viruses are typically detectable at 4-6 days post transfection.
    b. Optional: check for viral genome release into the supernatant by qPCR.

Day 4-7

10. Harvest supernatant as passage 0 (“P0”) stocks.
    a. Optionally, store at -80 °C.
11. Passage rescued virus in Mv.1.Lu cells according to the “HCoV-OC43 propagation” protocol above.
    a. For generation of P1 stocks, P0 stocks are typically passaged “blind” (i.e. without titration)
    b. For viruses with wildtype fitness, infect a T175 flask with 100 µL P0 supernatant.

Cloning archival fragments in pCLVR plasmids and propagation in *E. coli*

HCoV-OC43 fragments can be stably incorporated into the archival vector pCLVR. Linearisation primer pairs A1 and B1 allow vector linearisation by BsaI or BsmBI digestions, respectively.

Linearisation primers

pCLVRBSA1Fw	GGAGACCCGATCGGCAGGT
pCLVRBSA1Rev	CGAGACCATGGCAGGTGTGAGG
pCLVRBSMB1Fw	CGAGACGGCCGGAGACC
pCLVRBSMB1Rev	GGAGACGTGCTAGCGAGACC

Archiving Primers

B1_OC1Fw	GTCTCGCTAGCACGTCTCCATTGTGAGCGATTTGCGTGCG
B1_OC1Rev	CGGGTCTCCGGCCGTCTCGTAACTTTAGCACCTACACCTTCAAGCTCC
B1_OC2Fw	GTCTCGCTAGCACGTCTCCGTGGTGATTGATGCCATAGAAGAGAAACTTTCT
B1_OC2Rev	CGGGTCTCCGGCCGTCTCGGCTTTAATAATACGCTGAGTATAATACTTACCTCCGTCA
B1_OC3Fw	GTCTCGCTAGCACGTCTCCGAGTATAAACCTGACTTGTCACAATATTATTGTGACGG
B1_OC3Rev	CGGGTCTCCGGCCGTCTCGGGCGTATAACACTGCACTCCATCAG
A1_OC3Fw	CCTCACACCTGCCATGGTCTCGGAGTATAAACCTGACTTGTCACAATATTATTGTGACGG
A1_OC3Rev	CACCTGCCGATCGGGTCTCCGGCGTATAACACTGCACTCCATCAG
A1_OC4Fw	CCTCACACCTGCCATGGTCTCGGATATGGTTATCATGTGTTGCACTTTATTACACATGC
A1_OC4Rev	CACCTGCCGATCGGGTCTCCTAGAAGCAACATGCAAATGTTGCAAGCA
A1_OC5Fw	CCTCACACCTGCCATGGTCTCGCAAAATTGACTGATGTCAAATGTGCTAATGTCGTC
A1_OC5Rev	CACCTGCCGATCGGGTCTCCAAACAAACAACAACTGCTTAATGTCCACCATG
A1_OC6Fw	CCTCACACCTGCCATGGTCTCGGCTATTACTGATTATAATTATTATAAGTATAATTTGCCCACCATG
A1_OC6Rev	CACCTGCCGATCGGGTCTCCCTGACTATTATATGGGCTAATAAAAACAGCTTTATGCCAC
A1_OC7Fw	CCTCACACCTGCCATGGTCTCGGATTAATAAGTTTTTGAAGGCTAACCCTTTGTGGC
A1_OC7Rev	CACCTGCCGATCGGGTCTCCAAAGTCATAACTTTCTCATCGTTACACCAAAGCATAA
B1_OCF7Fw	GTCTCGCTAGCACGTCTCCGCCGATTAATAAGTTTTTGAAGGCTAACCCTTTGTGGC
B1_OC7Rev	CGGGTCTCCGGCCGTCTCGAAAGTCATAACTTTCTCATCGTTACACCAAAGCATAA
B1_OC8Fw	GTCTCGCTAGCACGTCTCCGCCAGTTAATGTTAATGTTGATTTTAAAGATTTTCAGTTTATGCTTTGG
B1_OC8Rev	CGGGTCTCCGGCCGTCTCGCACACAAATGGATGAATCAGGGATATTAATATCAGCAG
B1_OC9Fw	GTCTCGCTAGCACGTCTCCGCCTCTCTACCCCTATGGCAGATG
B1_OC9Rev	CGGGTCTCCGGCCGTCTCGAAACCGACATCAGATAACTTTACTTTATCAAAAAGTAAATCCTC
B1_OC10Fw	GTCTCGCTAGCACGTCTCCCTTATGCTTCCAGTAGATCTGCTATAGAGGATTTACTTT
B1_OC10Rev	CGGGTCTCCGGCCGTCTCGCACCTTGTCCTTCTACAAACTCAAACTCCTTTC
B1_OC11Fw	GTCTCGCTAGCACGTCTCCTATTCTTGGTTCTCTGGAATTACTCAGTTTCAAAAGG
B1_OC11Rev	CGGGTCTCCGGCCGTCTCGGTGATTCTTCCAATTGGCCATAATTAACTT

Preparation of fragments

1. For each OC43 fragment use viral cDNA or a synthesised gene fragment as a template, and a single pair of archiving primers.
2. Assemble PCR reactions using a high-fidelity polymerase, e.g. SuperFi II:

**Reagent**	**Volume**
Template (10-50 ng)	<1ul
Fw and Rev primer (100 uM)	0.3ul each
H_2O	Up to 25ul
SuperFi II 2x Master Mix	25ul

3. Run the following protocol in a thermocycler to generate PCR fragments:

**Stage**	**Temperature**	**Time**
1. Initial Denaturation	98°C	3 minutes
**2. Denaturation**	**98°C**	10 seconds
**3. Annealing**	**60°C**	10 seconds
**4. Extension**	**72°C**	30 seconds per kb (see below)
5. Final extension	72°C	5 minutes

12-24x cycles 2-4

Fragment	Extension time
Single	1min 30s
Double	3min
OC10/11	2min
Linearised pCLVR	1min

4. Run 3-5 µL of the reaction on a 1% agarose gel to determine successful PCR. A single band should be observed for each reaction.
5. Purify PCR products (e.g. using Nucleospin Gel and PCR clean up kit) and elute in 20 µL nuclease-free water.
6. To assemble archive fragments, in a fresh 0.2ml PCR tube, add:
    a. 50 ng of a 3 kb fragment or 100 ng of a 6 kb fragment.
    b. 25 ng of linearised pCLVR.
7. Add an equivalent volume of NEB HiFi 2x master mix and mix by pipetting gently
8. Incubate at 50 °C for 1 hour, followed by heat inactivation at 65 °C for 15 minutes.

Transformation

9. Thaw 10 µL NEB Stable cells on ice.
10. Add 2 µL of HiFi reaction, and incubate on ice for 5-15 minutes.
11. Heat shock cells by placing on a 42 °C heat block or water bath for 30 seconds, then return to ice.
12. Incubate on ice for 1 minute then add 100 µL SOC or other recovery medium.
13. Incubate at 37 °C shaking for 1 hour.
14. Spread transformed cells on a plate of LB agar with 50 µg/mL Kanamycin.
15. Incubate plates at 37 °C overnight.
16. Screen colonies by colony PCR:
    a. Label colonies on the plate.
    b. Prepare 10 uL PCR master mix per colony to be tested, using the appropriate amplification primers for the fragment.
    c. Using a pipette tip, gently dab the colony to pick up a small number of cells, then transfer this into the corresponding PCR tube and mix briefly.
    d. Amplify fragments with the same PCR parameters as above, for up to 30 cycles.
        i. n.b. pCLVR contains an mRFP1_Magenta chromoprotein, which is excised upon linearisation. Colonies transformed with residual circular vector will be pink in colour.
17. Transfer positive colonies into 5-50 mL LB broth + 50 µg/ml kanamycin.
18. Grow overnight at 37 °C, shaking at 225 rpm.
19. Purify plasmids by mini- or midiprep.
20. Send for full plasmid sequencing.

Amplicon sequencing of HCoV-OC43

Reagents:

- IPEGAL
- NaCl
- RNASIN (Promega, N2111)
- SuperFi II DNA polymerase 2x master mix
- Superscript VILO cDNA synthesis kit (Thermofisher, 11754050)

Optionally, use direct Lysis buffer, adapted from Direct Lysis RT-qPCR of SARS-CoV-2 in Cell Culture Supernatant Allows for Fast and Accurate Quantification - PMC

Direct lysis buffer can be used as an alternative to Trizol for inactivation of virus and cDNA synthesis without RNA purification. Amplicon generation and qPCR can be performed on cDNA from directly lysed samples. For 10 mL prepared direct lysis buffer:

Reagent	Volume
Nuclease-free water	9.35 mL
5M NaCl	300 µL
IGEPAL CA-630	250 µL
1M Tris-HCl	100 µL

Final concentration: NaCl 150 mM, Tris-HCl 10 uM, IGEPAL 2.5%

Store at 4 °C. Prior to use, take an aliquot, and add 1:1000 RNASIN then use immediately.

Sequencing primers

Primer name	Sequence
OC43Seq1Fw	ATTGTGAGCGATTTGCGTGCG
OC43Seq1Rev	TTTCTGCATCACTGCCAAAAC
OC43Seq2Fw	TAATGTTTATATGGCCAAGGCTGG
OC43Seq2Rev	GCAAATAAAAATCACTAAAAATTACATTGGC
OC43Seq3Fw	TACAGGTGATGTGGTGTTGGC
OC43Seq3Rev	GTCACTGTAAGAACAAGCATACAACC
OC43Seq4Fw	AACGTGCTTTTGGTGATTACACC
OC43Seq4Rev	GAGACGGGCATCTACACTCG
OC43Seq5Fw	TTCAGGTTGTTAATAGTGGTCCAG
OC32Seq5Rev	GCACTTAAATTAGCTACTGAATGAGAGG
OC43Seq6Fw	AGTGTTATTTTAAGTACTTGTGATGGAC
OC43Seq6Rev	GTCGAATTGGCTGAAGATGACATC
OC43Seq7Fw	TGCTAAGCAGGTTGATTATGTACC
OC43Seq7Fw	GATTAATATCATAAACACCAACATAGTTGG
OC43Seq8Fw	CTGGTAAAATATCATCTAAAGCTGGC
OC43Seq8Rev	CCACAGAAATTTATCCTAGATGATTGGC
OC43Seq9Fw	ATTAGGGACCTCATTTGTGTGC
OC43Seq9Rev	TTTTTTTTGTGATTCTTCCAATTGGC

This generates 9 PCR amplicons of ~3.5 kb which overlap by ~800 bp.

Protocol

1. Extract RNA from infected cells or supernatants:
    a. For direct lysis, mix supernatant 1:1 with direct lysis buffer.
    b. Invert the tube to mix then incubate at room temperature for 20 mins.
    c. Store lysed supernatant at -80 °C. If using same day, perform at least 1 freeze-thaw cycle to promote lysis of viral particles.

2. Perform reverse transcription, e.g. using Superscript VILO:

Reagent	Volume
Nuclease-free water	11.5 µL
5x VILO Master Mix	4 µL
10x SuperScript Enzyme	2 µL
RNA or lysed supernatant	2.5 µL

Temperature (°C)	Time
25	10 min
42	1 hour
85	5min
4	forever

3. Optional: cDNA may be stored at -20ºC.
4. Prepare a separate 10 µL PCR reaction for each amplicon pair:

Reagent	Volume
SuperFi II 2x Master Mix	5 µL
FwPrimer (10uM)	1 µL
RevPrimer (10uM)	1 µL
cDNA	1 µL
Nuclease free water	2 µL

5. Run the following protocol in a thermocycler to generate PCR fragments:

Stage	Temperature	Time
1. Initial Denaturation	98ºC	3min
**2. Denaturation**	**98ºC**	**10s**
**3. Annealing**	**60ºC**	**10s**
**4. Extension**	**72ºC**	**2.5 minutes**
5. Final extension	72ºC	5 minutes

20-35x cycles 2-4

6. Take 1 µL of each sample and run on a 1% agarose gel to confirm successful amplicon generation.
7. Pool PCR amplicons and purify (e.g. using Nucleospin Gel and PCR clean up kit).
8. Prepare amplicons for nanopore sequencing or send to external sequencing service (e.g. Full Circle).
